# Supplementary material for: Functional roles of Aves class-specific cis-regulatory elements on macroevolution of bird-specific features
Source: Nat Commun. 2017 Feb 6;8:14229. doi: 10.1038/ncomms14229 (PMC5473641; doi:10.1038/ncomms14229)
Supplement: Supplementary Information — Supplementary Figures, Supplementary Tables, and Supplementary References [file ncomms14229-s1.pdf]

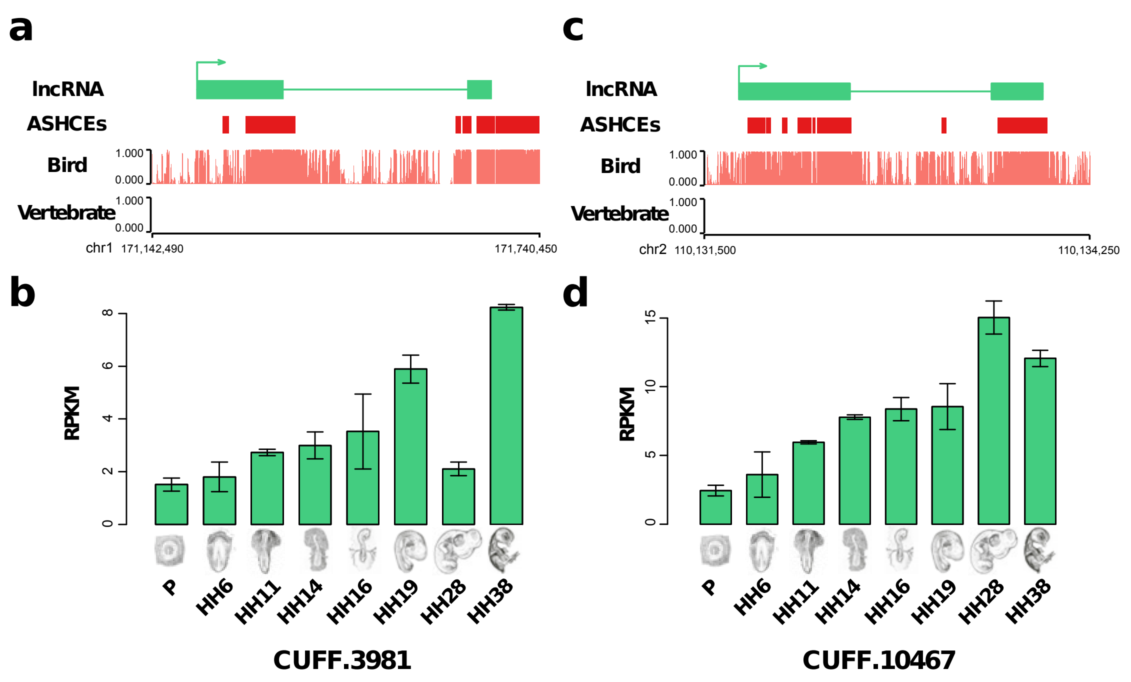

Supplementary Figure 1. Two lncRNAs overlapping with ASHCEs and their expression profiles across different developmental stages. (**a**) The gene structure of lncRNA CUFF.3981 and the conservation scores in birds and vertebrates. (**b**) Expression profile of CUFF.3981 at different developmental stages. (**c**) The gene structure of lncRNA CUFF.10467 and the conservation scores in birds and vertebrates. (**d**) Expression profile of CUFF.10467 at different developmental stages. Expression values of two replicates were used, and the error bars indicate the standard deviations.

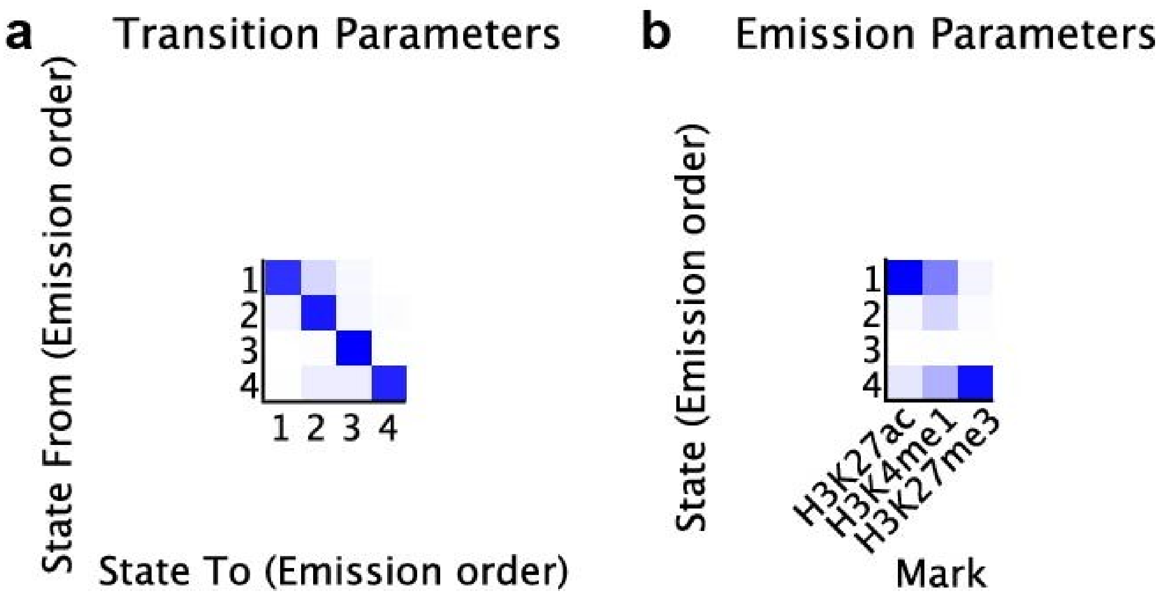

Supplementary Figure 2. Heatmaps of model learning parameters for 4 states in the *ChromHMM* analysis. (a) heatmap for transition parameters for 4 states (E1-E4). **(b)** heatmaps for emission parameters.

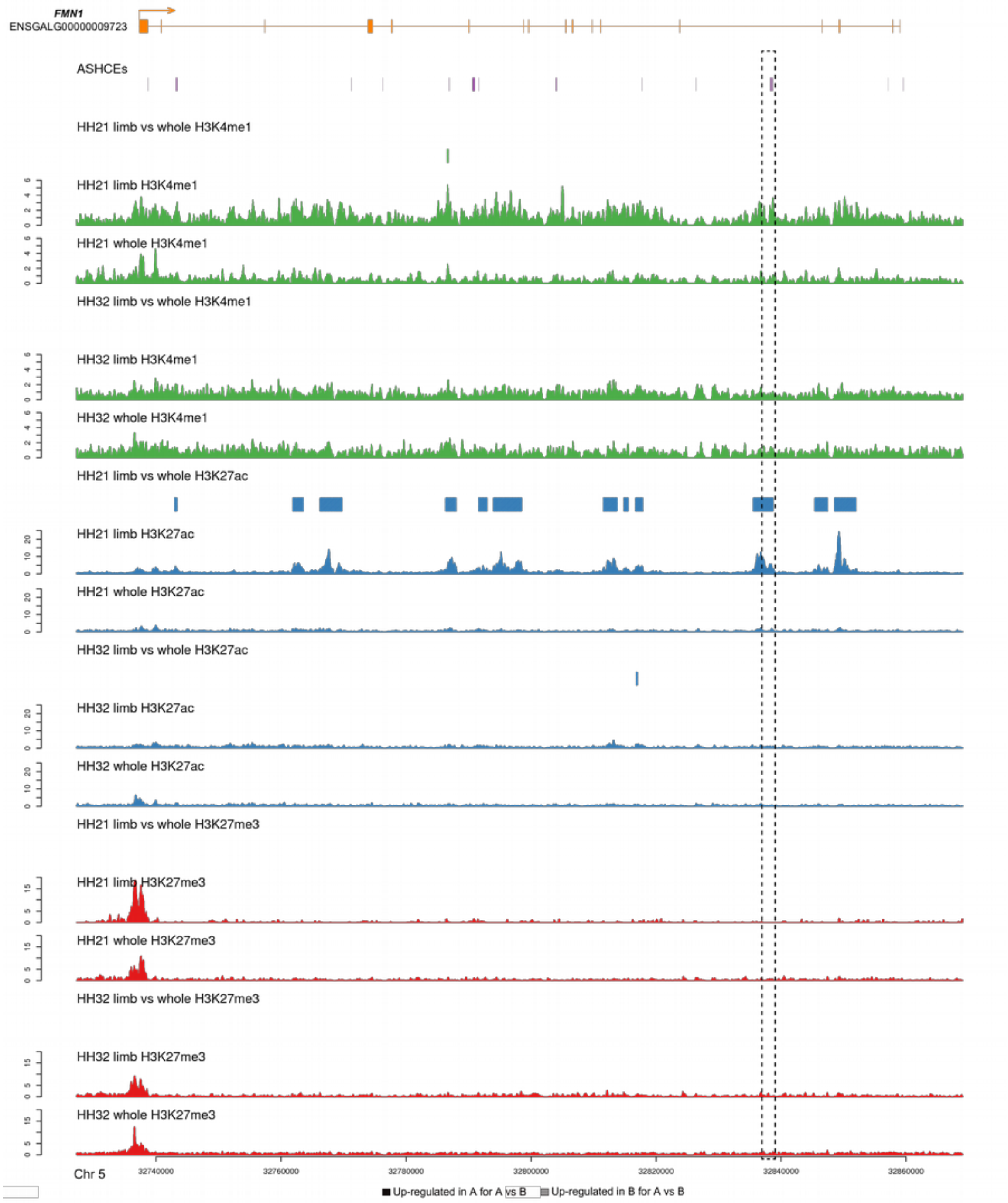

FMN1
ENSGALG00000009723
ASHCEs
HH21 limb vs whole H3K4me1
HH21 limb H3K4me1
HH21 whole H3K4me1
HH32 limb vs whole H3K4me1
HH32 limb H3K4me1
HH32 whole H3K4me1
HH21 limb vs whole H3K27ac
HH21 limb H3K27ac
HH21 whole H3K27ac
HH32 limb vs whole H3K27ac
HH32 limb H3K27ac
HH32 whole H3K27ac
HH21 limb vs whole H3K27me3
HH21 limb H3K27me3
HH21 whole H3K27me3
HH32 limb vs whole H3K27me3
HH32 limb H3K27me3
HH32 whole H3K27me3
Chr 5
32740000
32760000
32780000
32800000
32820000
32840000
32860000
Up-regulated in A for A vs B
Up-regulated in B for A vs B

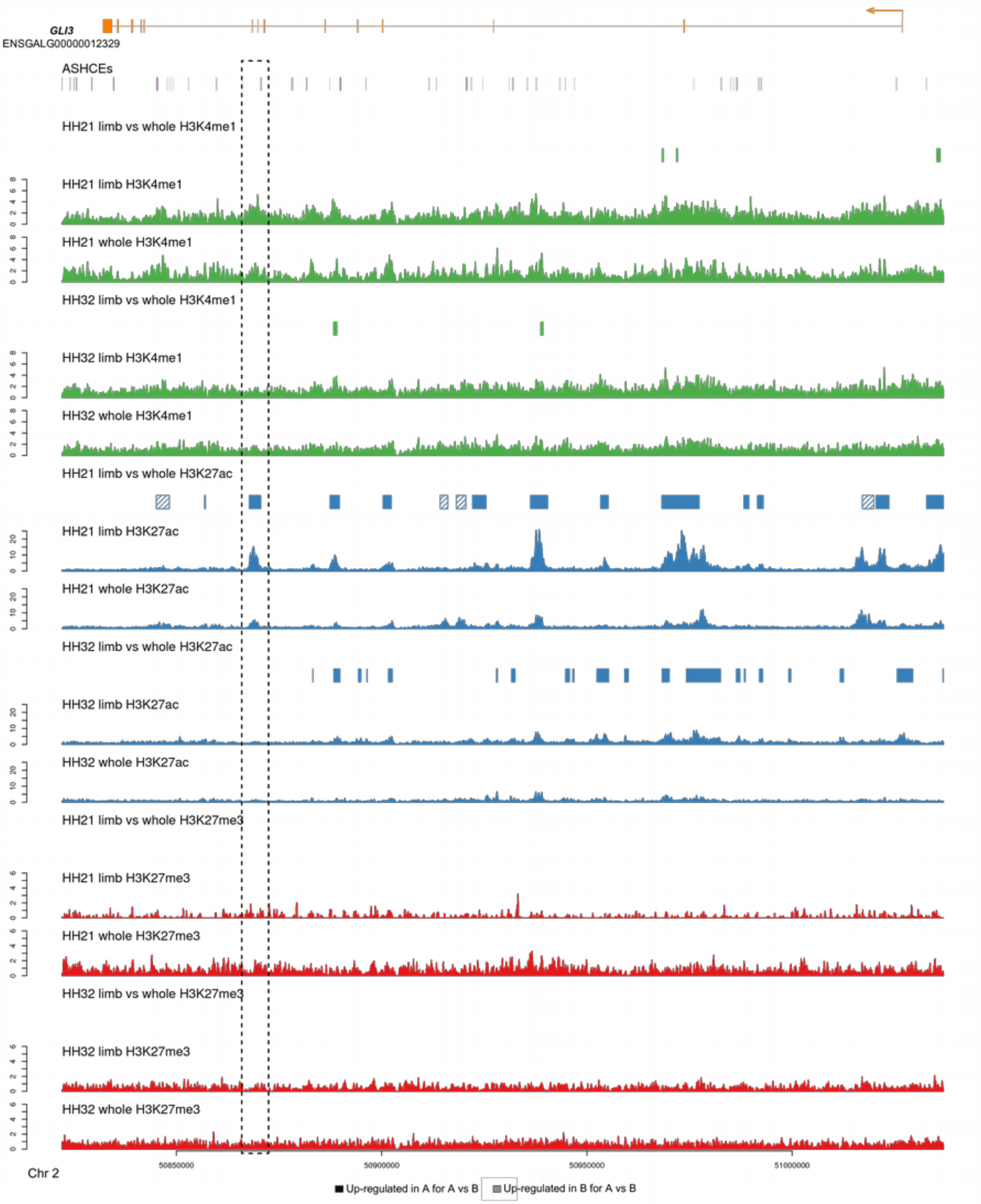
GLI3
ENSGALG00000012329
ASHCEs
HH21 limb vs whole H3K4me1
HH21 limb H3K4me1
HH21 whole H3K4me1
HH32 limb vs whole H3K4me1
HH32 limb H3K4me1
HH32 whole H3K4me1
HH21 limb vs whole H3K27ac
HH21 limb H3K27ac
HH21 whole H3K27ac
HH32 limb vs whole H3K27ac
HH32 limb H3K27ac
HH32 whole H3K27ac
HH21 limb vs whole H3K27me3
HH21 limb H3K27me3
HH21 whole H3K27me3
HH32 limb vs whole H3K27me3
HH32 limb H3K27me3
HH32 whole H3K27me3
Chr 2
50850000
50900000
50950000
51000000
Up-regulated in A for A vs B
Up-regulated in B for A vs B

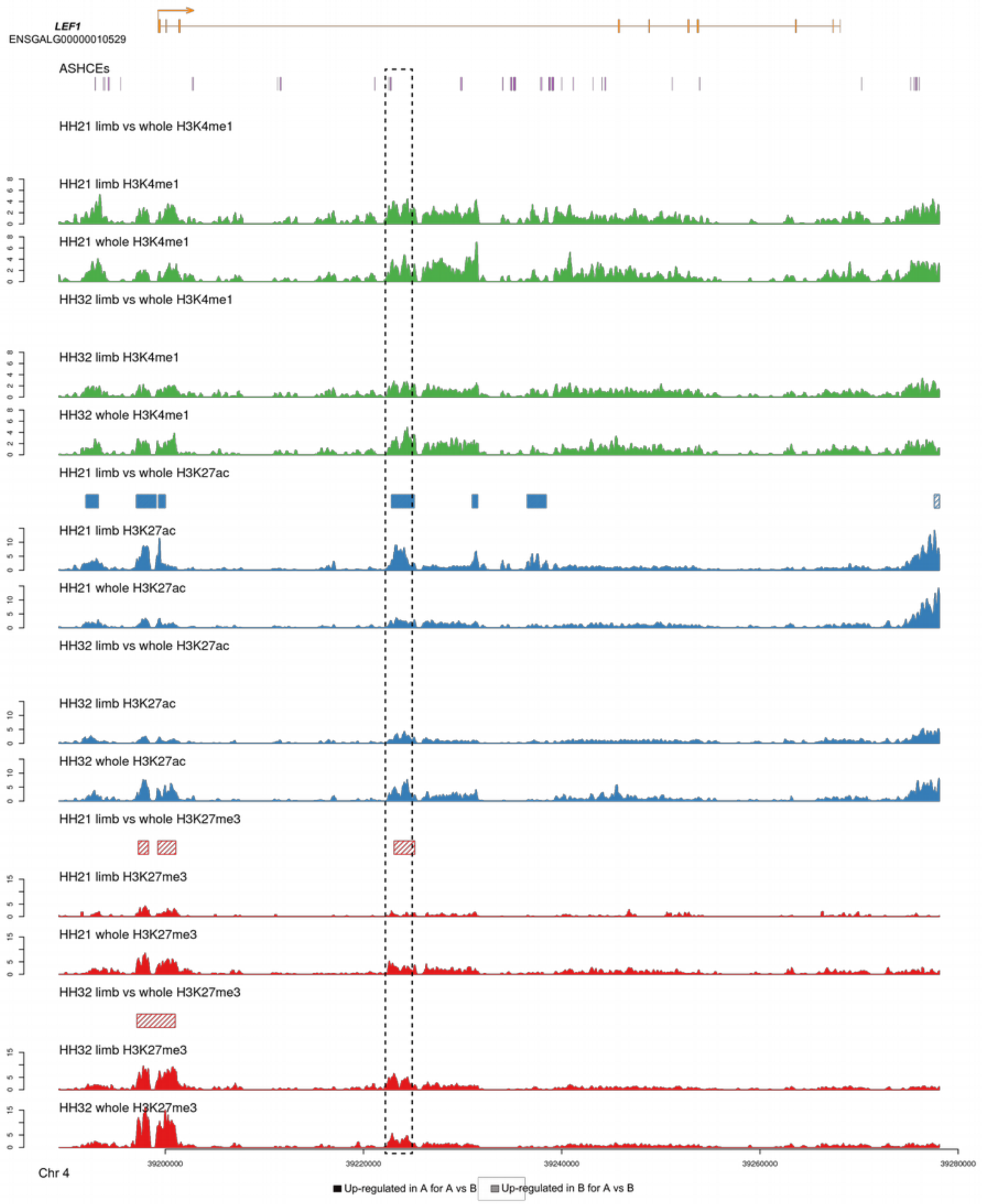
LEF1
ENSGALG00000010529
ASHCEs
HH21 limb vs whole H3K4me1
HH21 limb H3K4me1
HH21 whole H3K4me1
HH32 limb vs whole H3K4me1
HH32 limb H3K4me1
HH32 whole H3K4me1
HH21 limb vs whole H3K27ac
HH21 limb H3K27ac
HH21 whole H3K27ac
HH32 limb vs whole H3K27ac
HH32 limb H3K27ac
HH32 whole H3K27ac
HH21 limb vs whole H3K27me3
HH21 limb H3K27me3
HH21 whole H3K27me3
HH32 limb vs whole H3K27me3
HH32 limb H3K27me3
HH32 whole H3K27me3
Chr 4
39200000
39220000
39240000
39260000
39280000
Up-regulated in A for A vs B
Up-regulated in B for A vs B

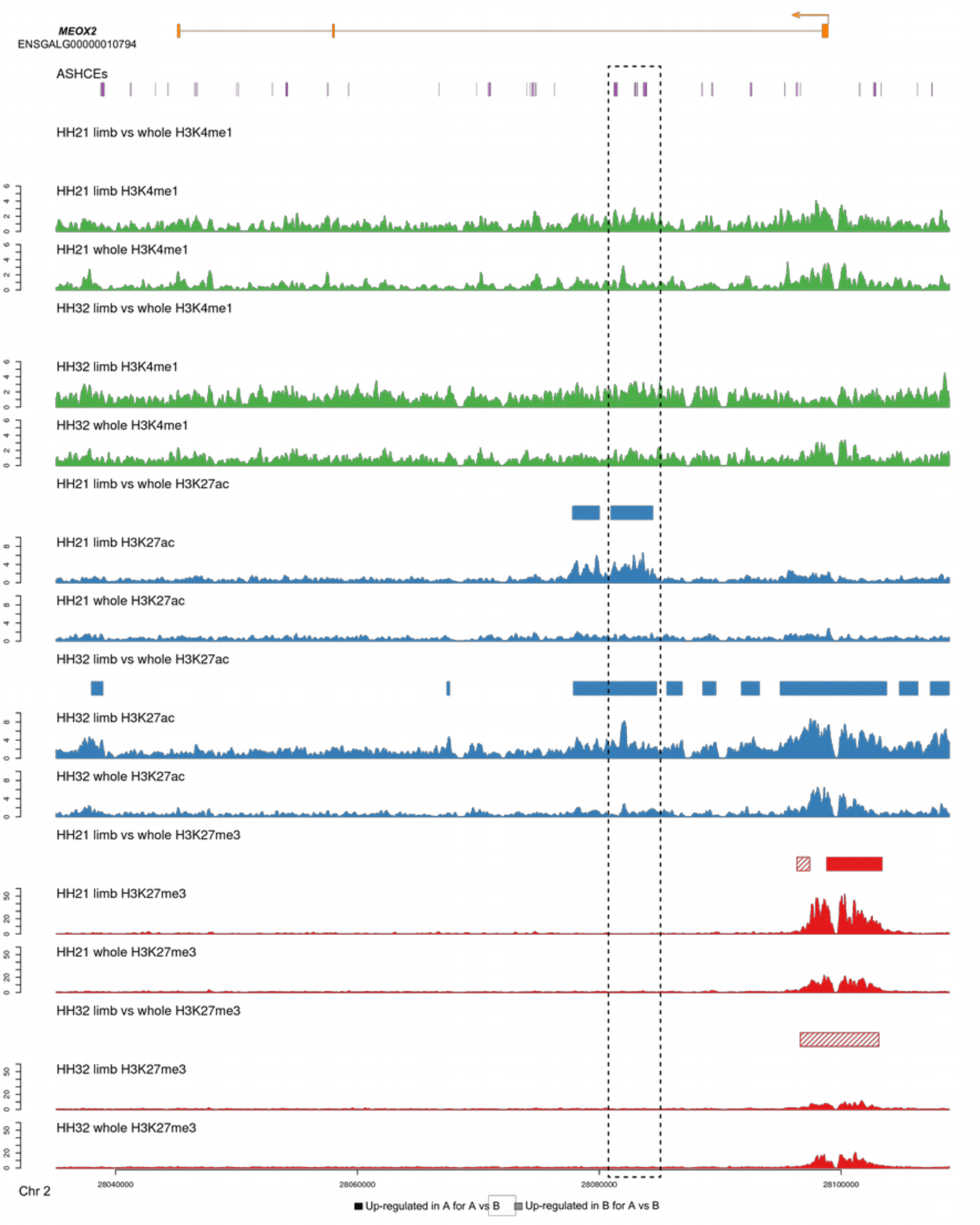
MEOX2
ENSGALG00000010794
ASHCEs
HH21 limb vs whole H3K4me1
HH21 limb H3K4me1
HH21 whole H3K4me1
HH32 limb vs whole H3K4me1
HH32 limb H3K4me1
HH32 whole H3K4me1
HH21 limb vs whole H3K27ac
HH21 limb H3K27ac
HH21 whole H3K27ac
HH32 limb vs whole H3K27ac
HH32 limb H3K27ac
HH32 whole H3K27ac
HH21 limb vs whole H3K27me3
HH21 limb H3K27me3
HH21 whole H3K27me3
HH32 limb vs whole H3K27me3
HH32 limb H3K27me3
HH32 whole H3K27me3
Chr 2
28040000
28060000
28080000
28100000
Up-regulated in A for A vs B
Up-regulated in B for A vs B

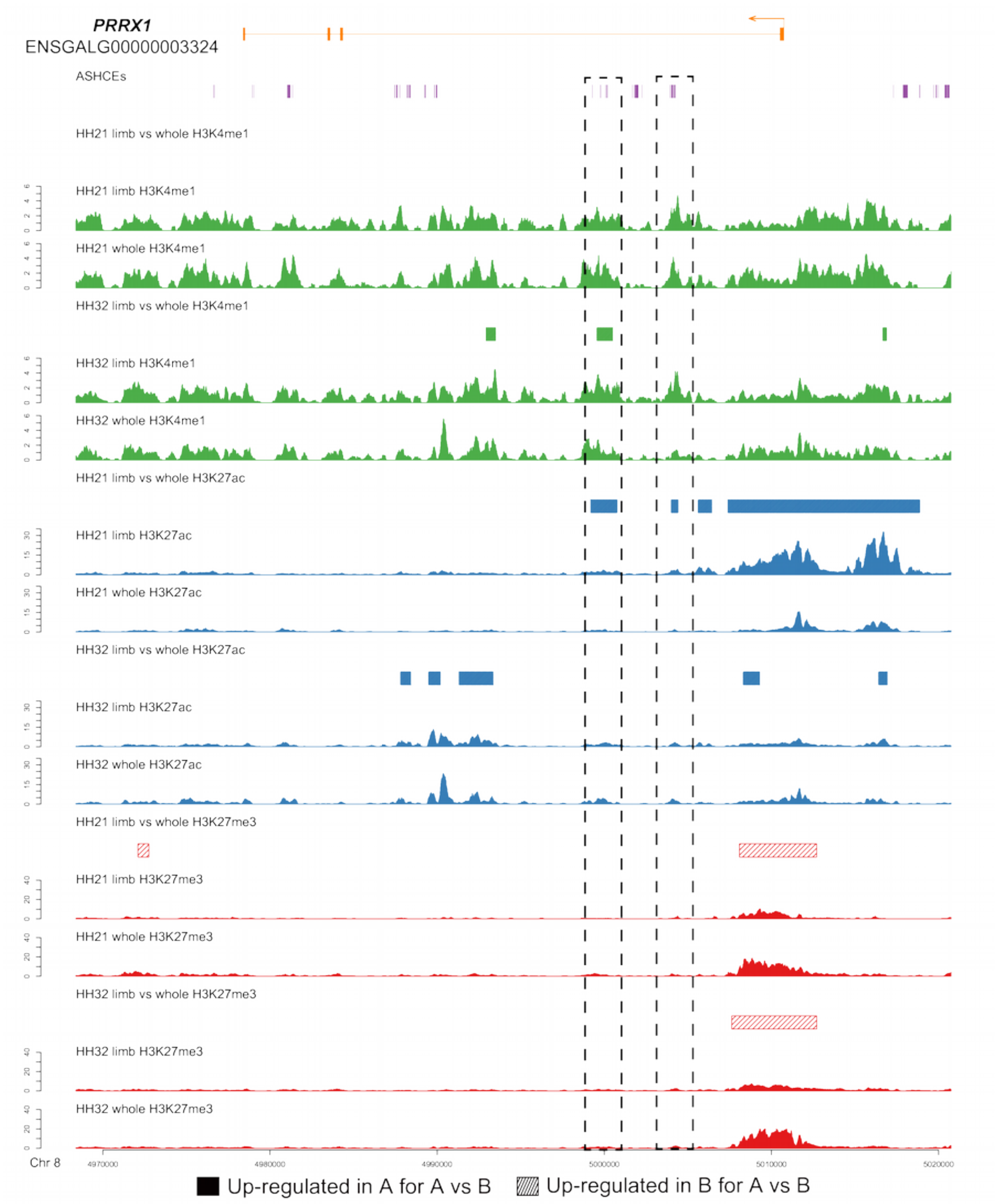

Supplementary Figure 3. Limb-related genes (based on GO annotation) with ASHCEs overlapping differential histone modification sites between limb and whole embryo samples. The dash-line boxes highlight the ASHCEs regions with differential histone modification sites. The signal tracks are plotted based on the average signal of two replicates of each condition.

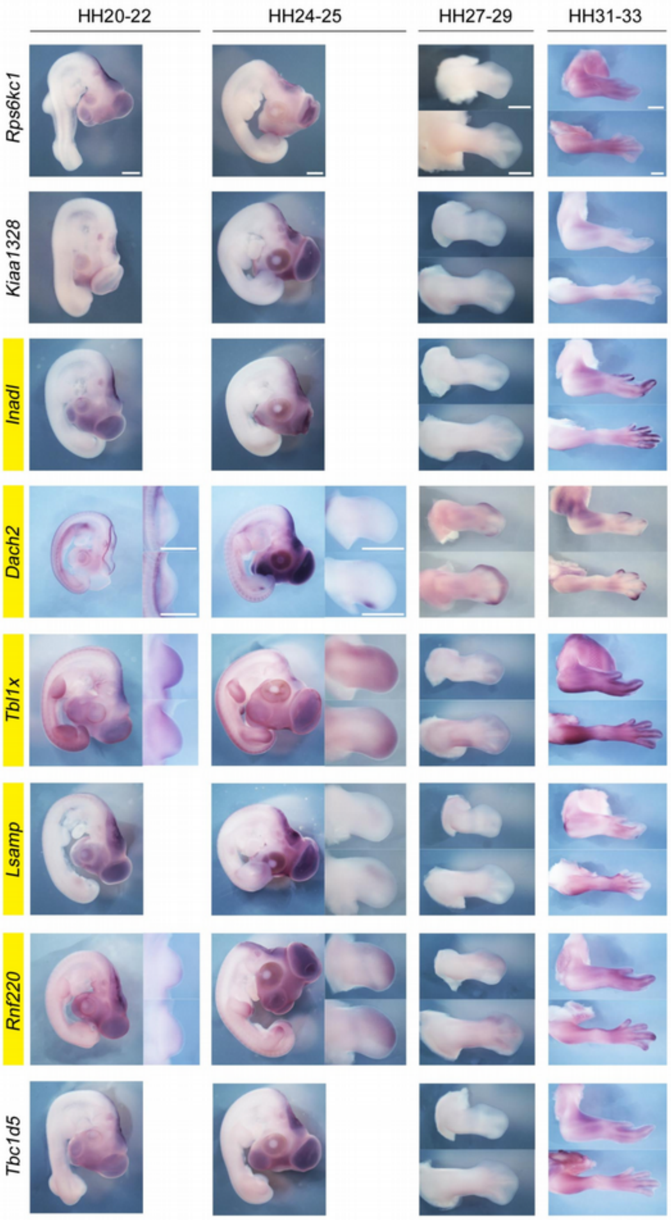
HH20-22
HH24-25
HH27-29
HH31-33
Rps6kc1
Kiaa1328
Inadl
Dach2
Tbl1x
Lsamp
Rnf220
Tbc1d5

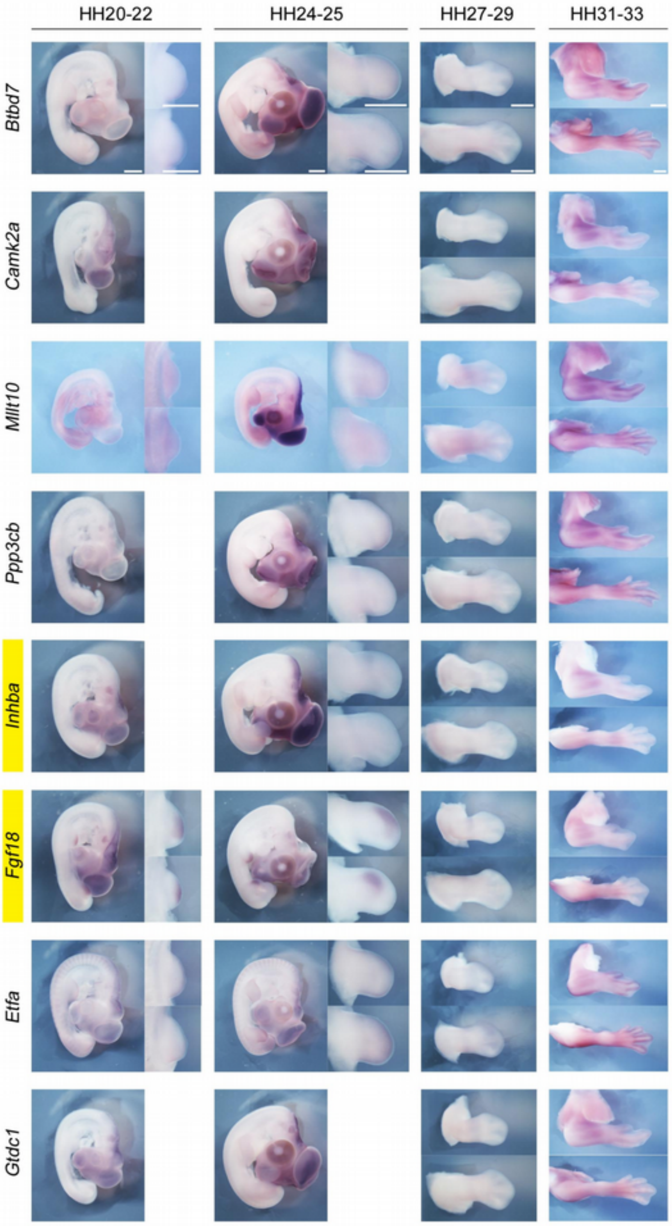
HH20-22
HH24-25
HH27-29
HH31-33
Btbd7
Camk2a
Mllt10
Ppp3cb
Inhba
Fgf18
Etfa
Gtdc1

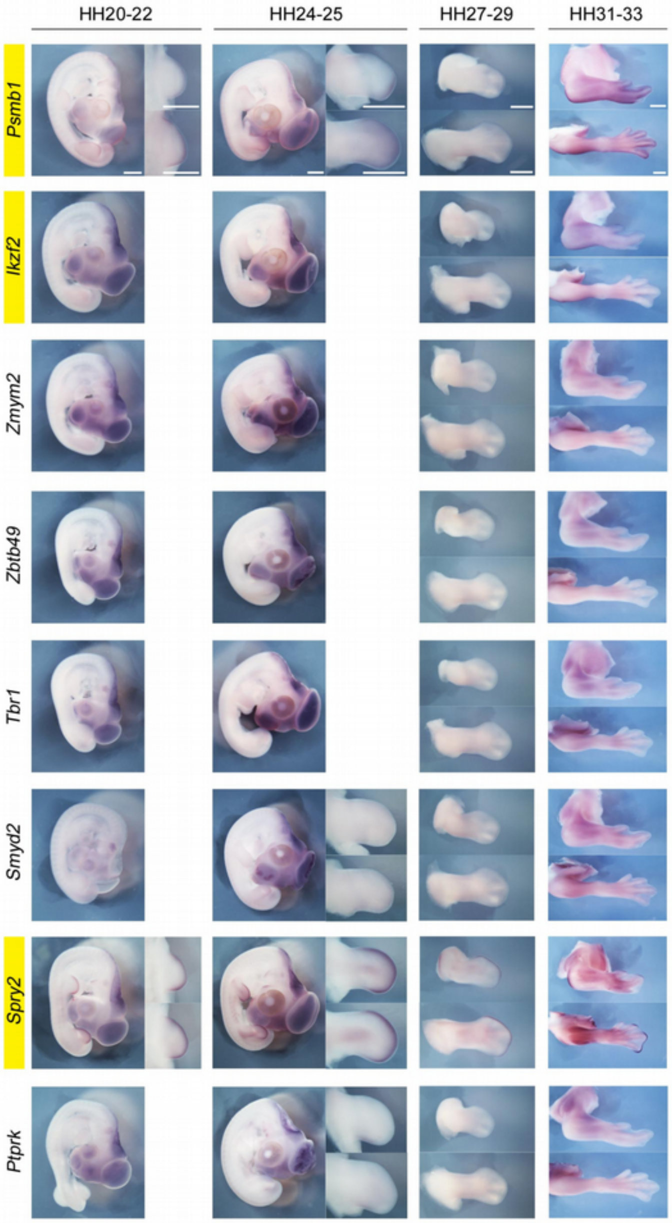
HH20-22
HH24-25
HH27-29
HH31-33
Psmb1
Ikzf2
Zmym2
Zbtb49
Tbr1
Smyd2
Spry2
Ptprk

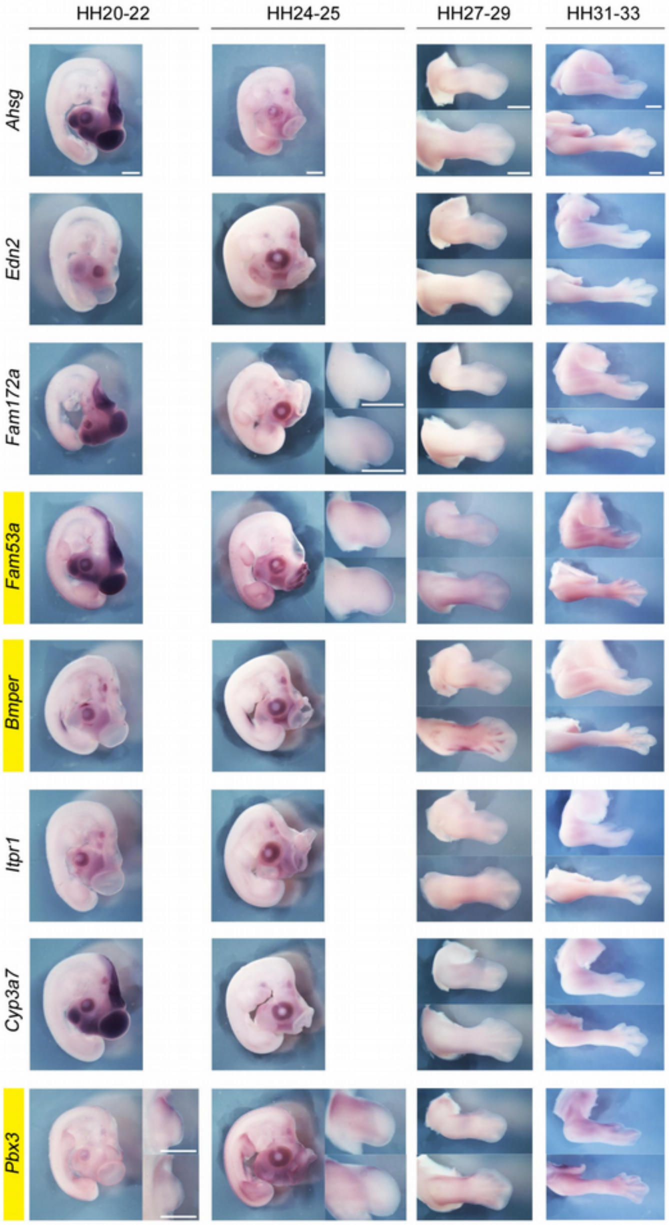
HH20-22
HH24-25
HH27-29
HH31-33
Ahsg
Edn2
Fam172a
Fam53a
Bmper
Itpr1
Cyp3a7
Pbx3

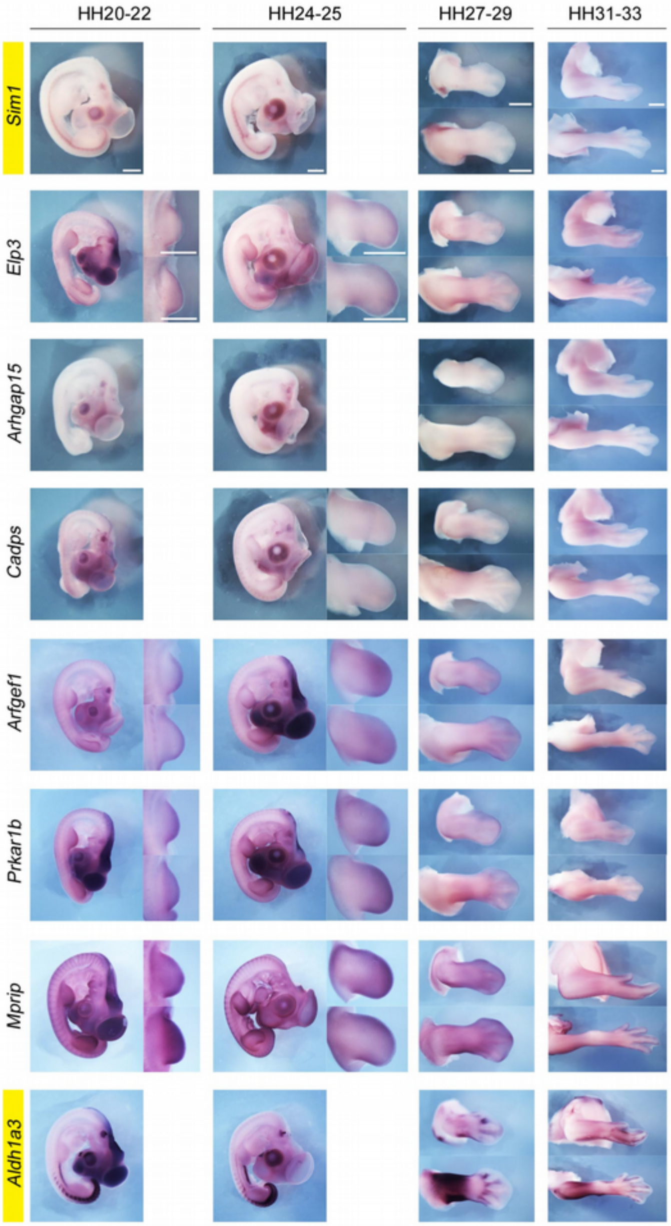
HH20-22
HH24-25
HH27-29
HH31-33
Sim1
Elp3
Arhgap15
Cadps
Arfgef1
Prkar1b
Mprip
Aldh1a3

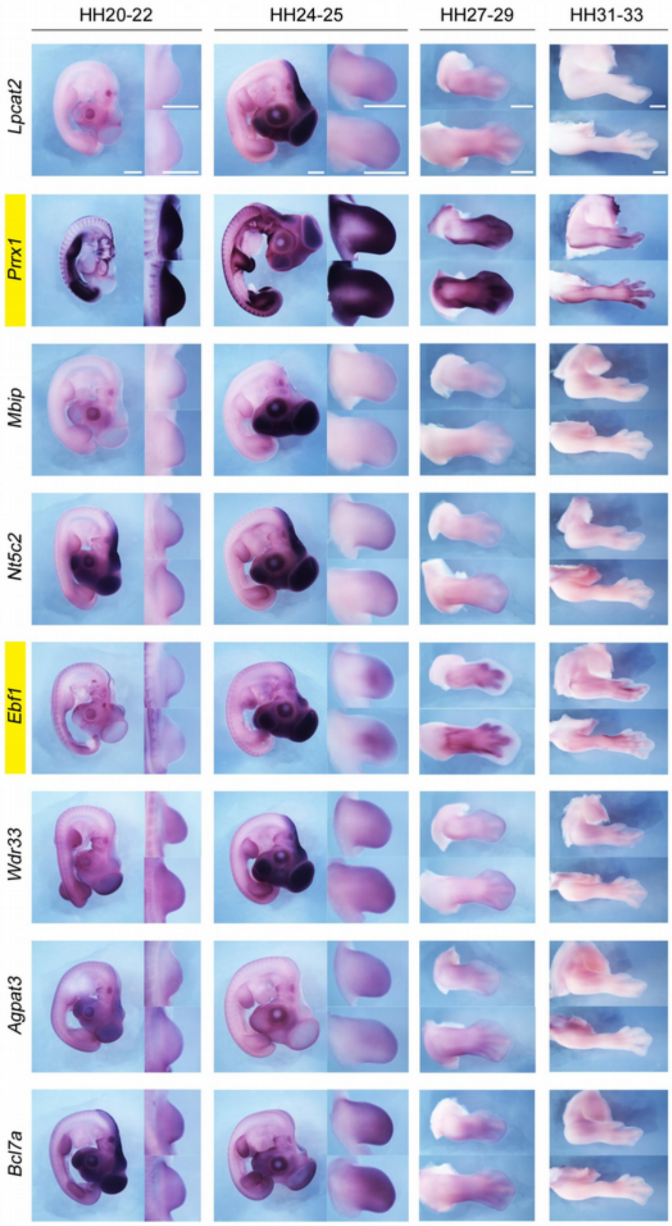
HH20-22
HH24-25
HH27-29
HH31-33
Lpcat2
Prrx1
Mbip
Nt5c2
Ebf1
Wdr33
Agpat3
Bcl7a

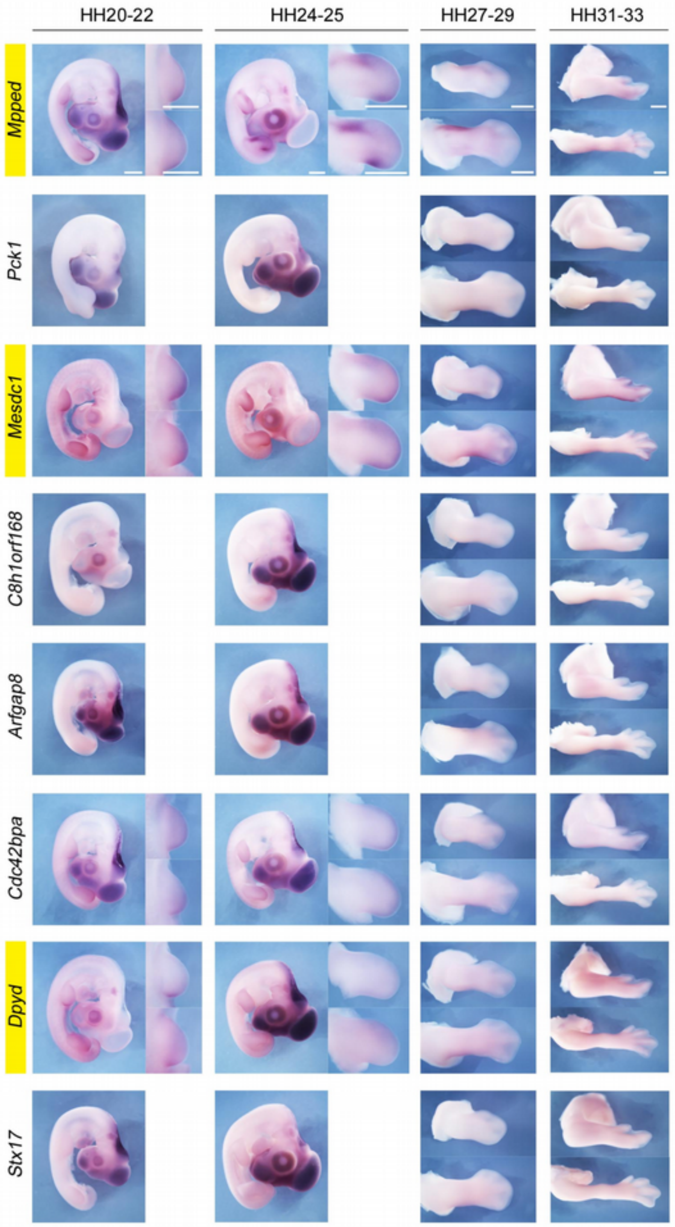
HH20-22
HH24-25
HH27-29
HH31-33
Mpped
Pck1
Mesdc1
C8h1orf168
Arfgap8
Cdc42bpa
Dpyd
Stx17

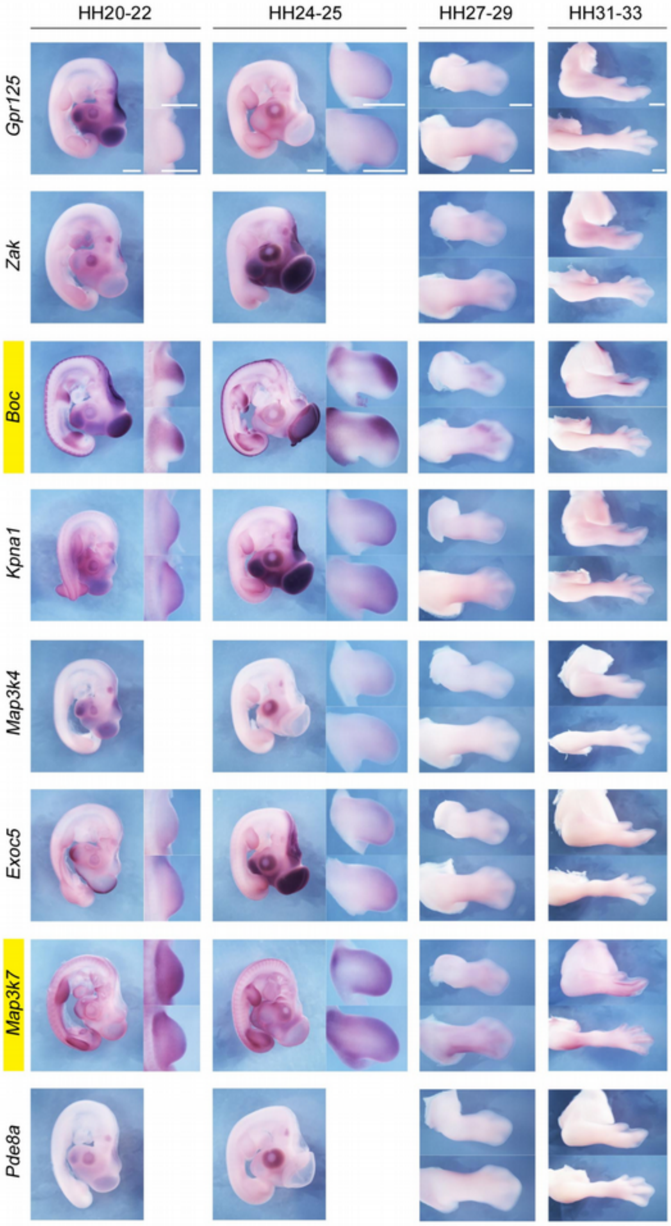
HH20-22
HH24-25
HH27-29
HH31-33
Gpr125
Zak
Boc
Kpna1
Map3k4
Exoc5
Map3k7
Pde8a

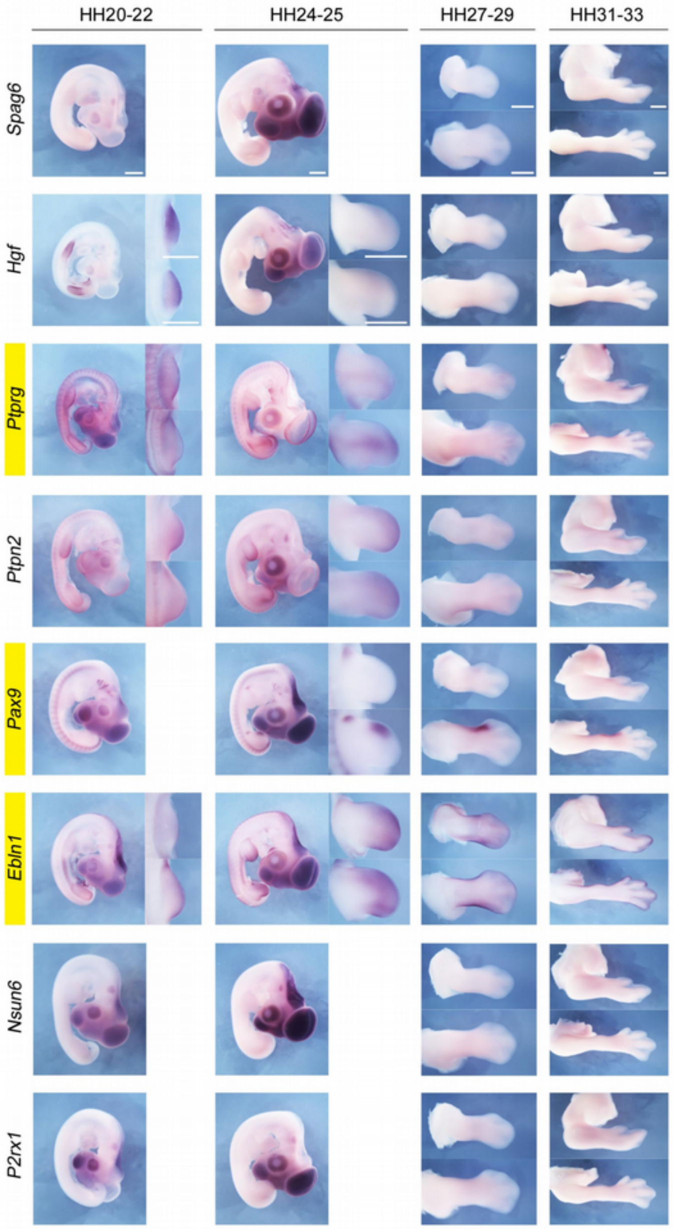
HH20-22
HH24-25
HH27-29
HH31-33
Spag6
Hgf
Ptprg
Ptpn2
Pax9
Ebln1
Nsun6
P2rx1

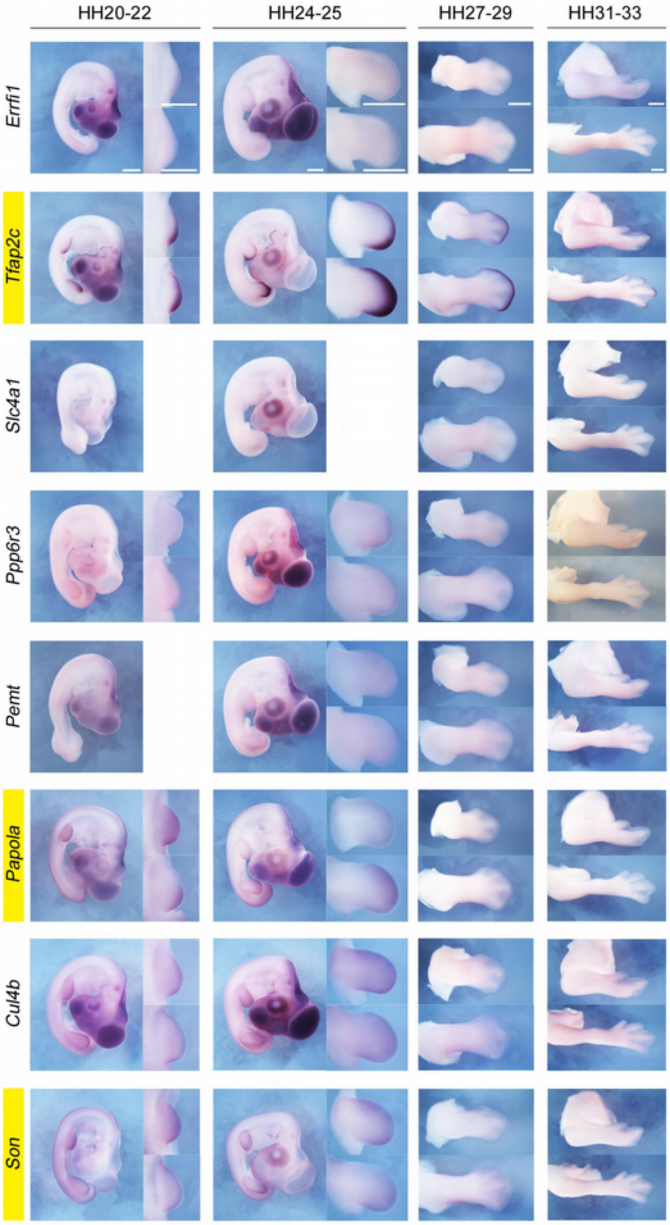
HH20-22
HH24-25
HH27-29
HH31-33
Errfi1
Tfap2c
Slc4a1
Ppp6r3
Pemt
Papola
Cul4b
Son

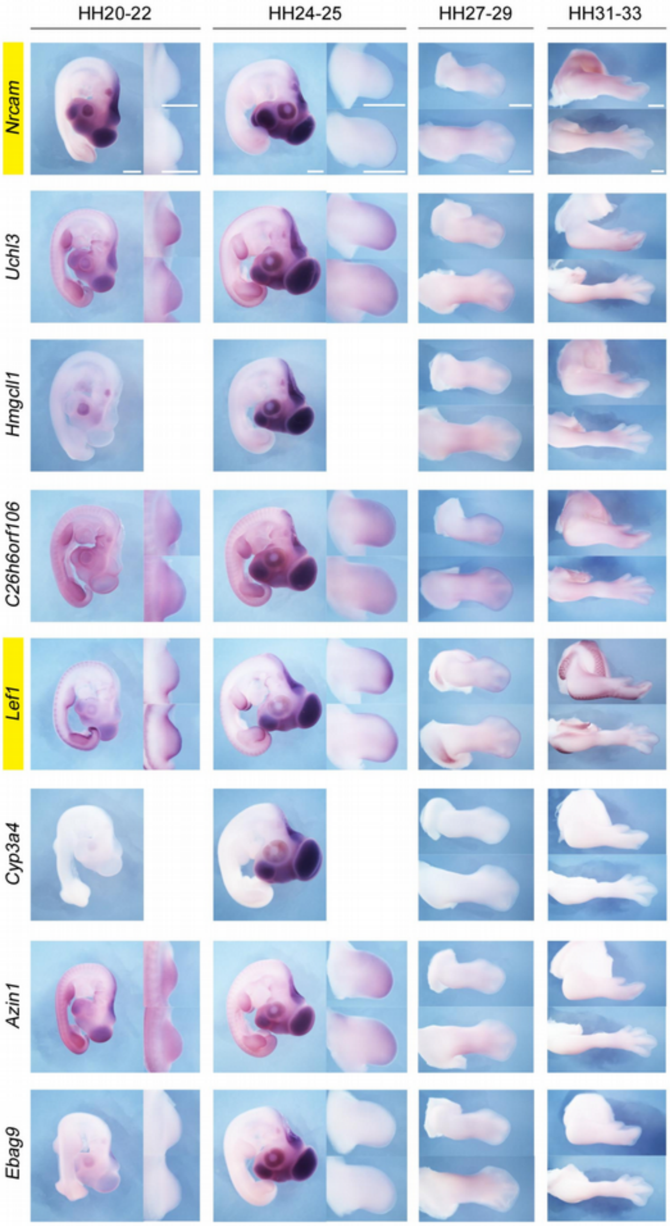

HH20-22
HH24-25
HH27-29
HH31-33
Nrcam
Uchl3
Hmgcll1
C26h6orf106
Lef1
Cyp3a4
Azin1
Ebag9

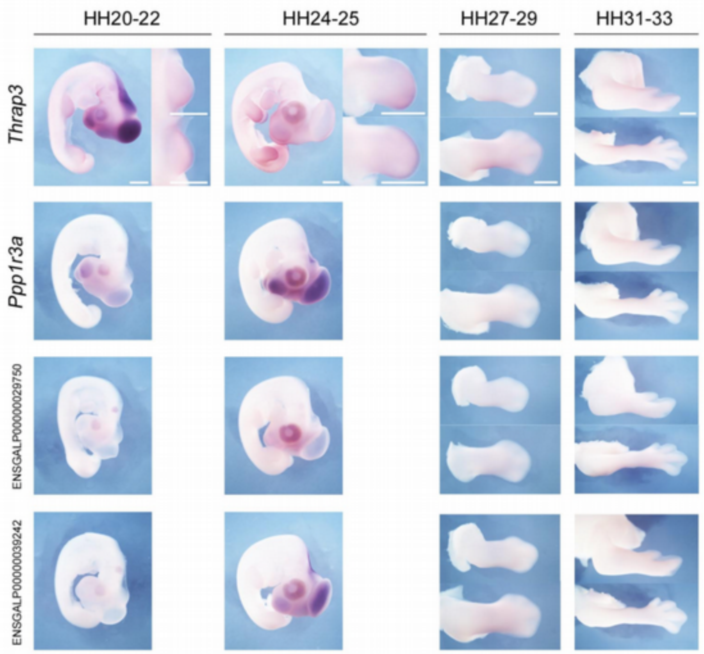

Supplementary Figure 4. *in situ* hybridization assay of ASHCEs-associated genes in the chicken embryo. A table showing expression profiles of 92 genes used for the first *in situ* expression screening with the developing chicken limb bud. Eight of 100 genes were not successfully cloned from chicken embryonic cDNA. The 100 genes were selected from an early version of top 500 gene list. Nine genes (*AGPAT3, BMPER, ITPR1, KIAA1328, MBIP, MPRIP, TBL1X, TFAP2C* and *ZMYM2*) were absent from the final version top 500 gene list after updating the filtering criteria, but still be included here for reference. Developmental stages examined were HH20- 22, 24-25, 27-29 and 31-33 as shown from the left to right columns for each gene. Fore- and hindlimbs at HH20-22 and 24-25 are magnified if there was any expression signal. Some pictures are flip-flopped horizontally to arrange the proximo-distal and antero-posterior axes. Thirty of the 92 ASHCEs-associated genes that showed localized and/or obvious expression in the chicken embryo (highlighted in yellow) were selected and further used for the second screening in the mouse (see **Supplementary Fig. 5**). Scale bars, 1 mm.

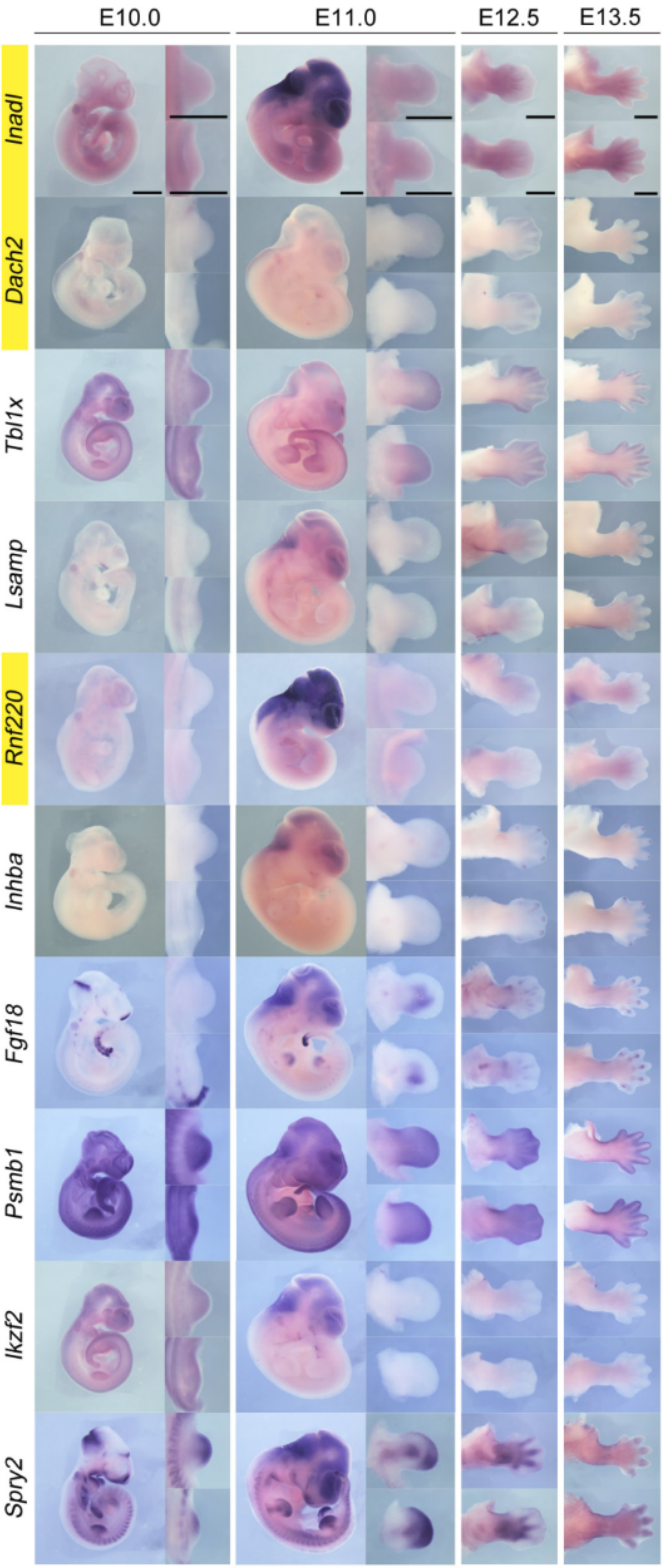
E10.0
E11.0
E12.5
E13.5
Inadl
Dach2
Tbl1x
Lsamp
Rnf220
Inhba
Fgf18
Psmb1
Ikzf2
Spry2

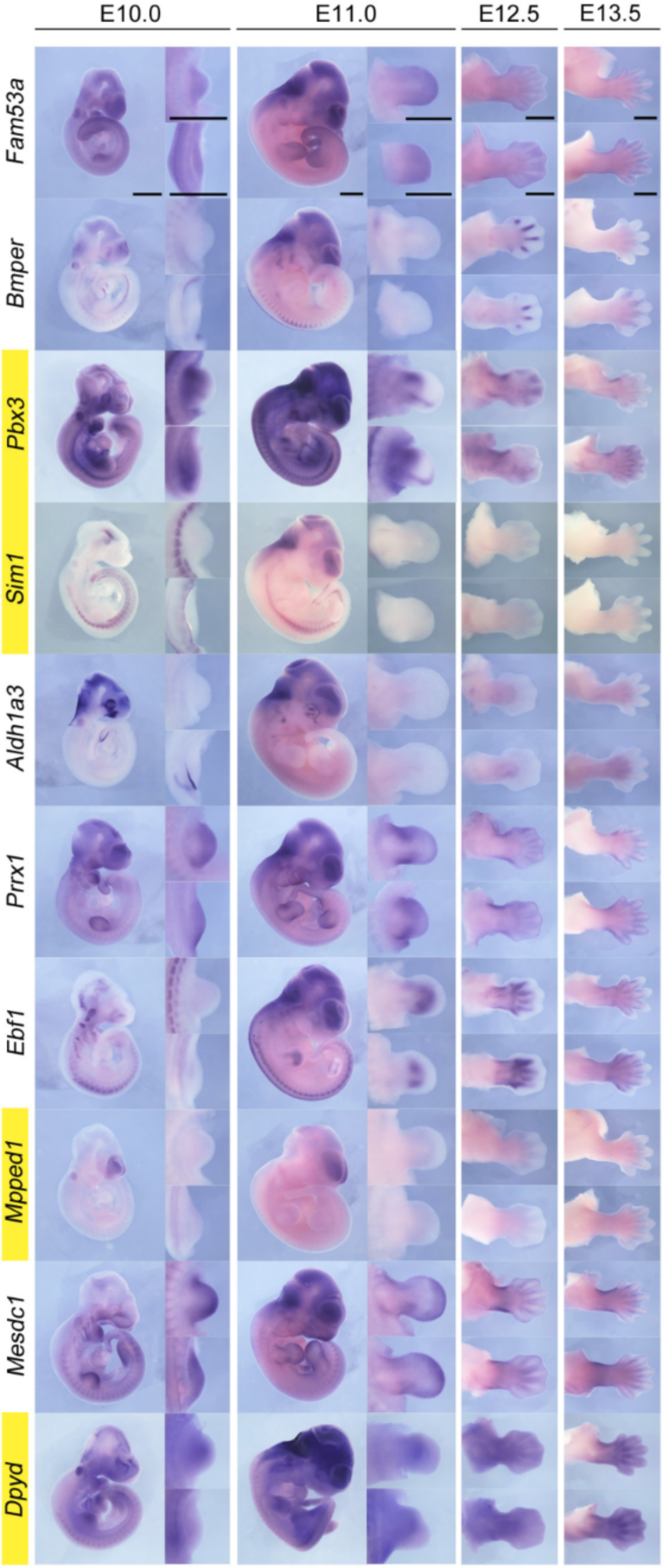
E10.0
E11.0
E12.5
E13.5
Fam53a
Bmper
Pbx3
Sim1
Aldh1a3
Prrx1
Ebf1
Mpped1
Mesdc1
Dpyd

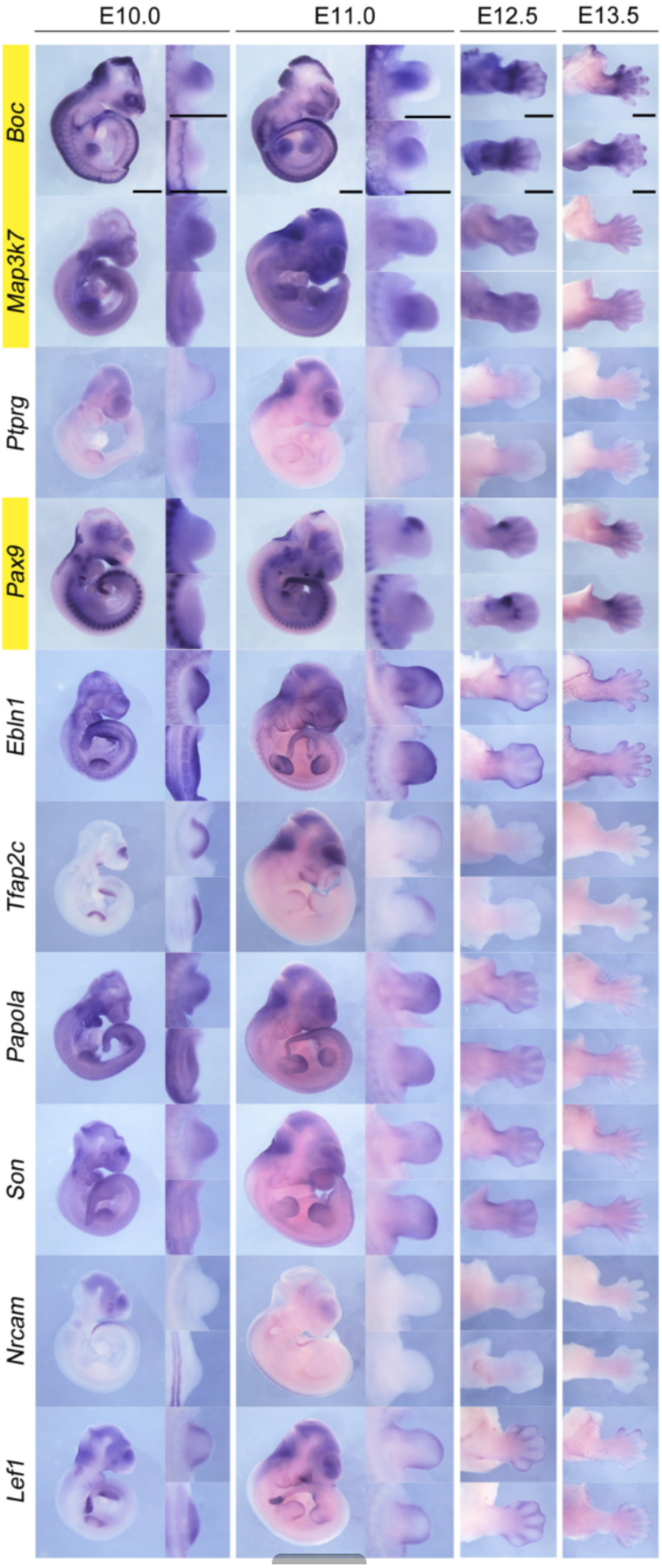
E10.0
E11.0
E12.5
E13.5
Boc
Map3k7
Ptprg
Pax9
Ebln1
Tfap2c
Papola
Son
Nrcam
Lef1

Supplementary Figure 5. *in situ* hybridization assay of the first-screened genes in the mouse embryo. A table showing expression profiles of 30 genes used for the second in situ expression screening with the developing mouse limb bud. Whole-mount *in situ* hybridization was performed for mouse orthologues of 30 ASHCEs-associated genes selected in the first screening (**Supplementary Fig. 4**). Mouse embryos at E10.0, E11.0, E12.5 and E13.5, stages approximately corresponding to those of chicken embryos, were analyzed and the results are shown from the left to right columns for each gene. Some pictures are flip-flopped horizontally to arrange the proximo-distal and antero-posterior axes. Ten of 30 genes that showed different expression patterns from those in the chicken embryo (highlighted in yellow) were selected and further used for the final screening in the gecko (see **Supplementary Fig. 6**). Scale bars, 1 mm.

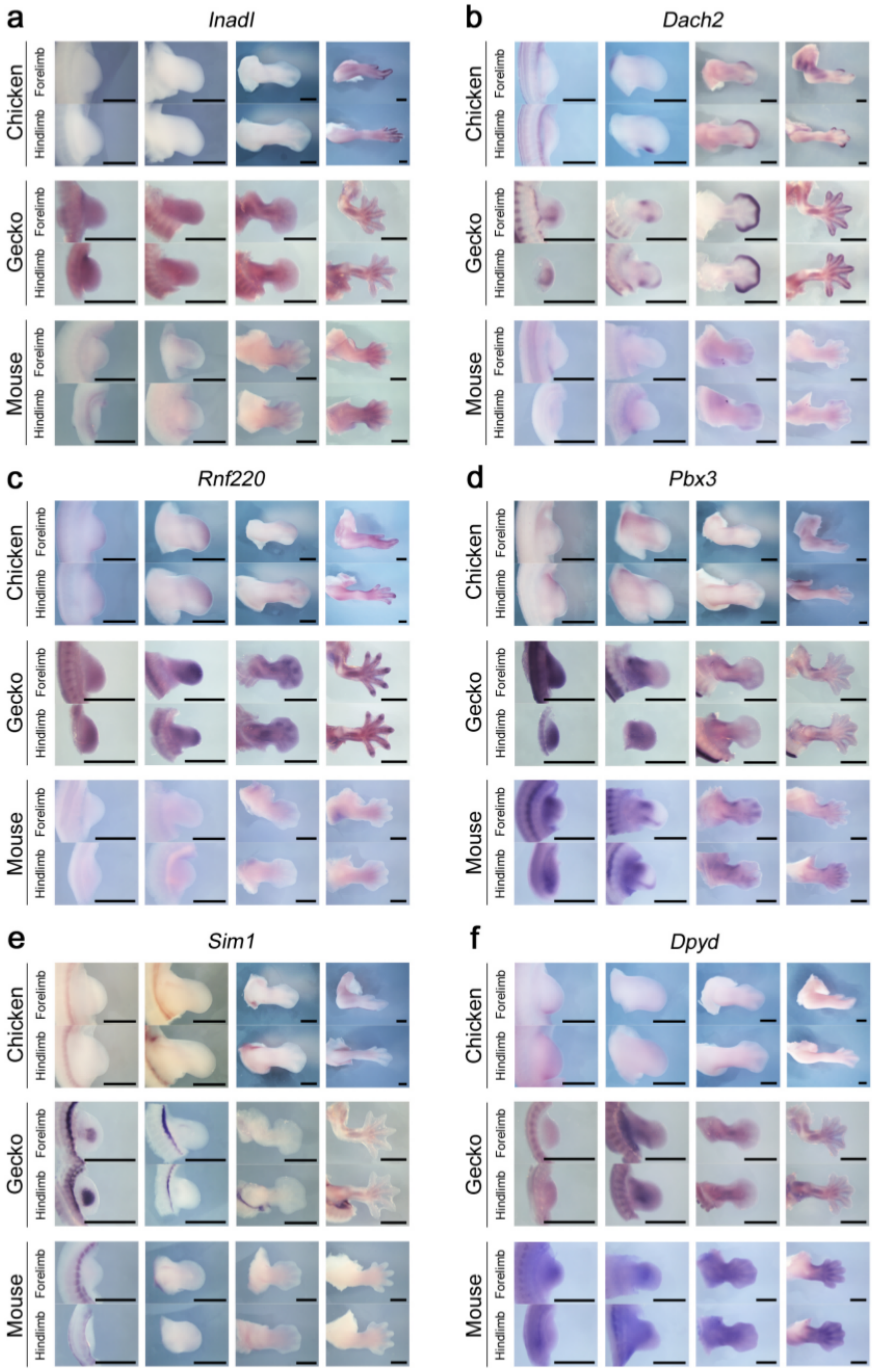

a
Inadl
b
Dach2
c
Rnf220
d
Pbx3
e
Sim1
f
Dpyd
Chicken
Gecko
Mouse
Forelimb
Hindlimb

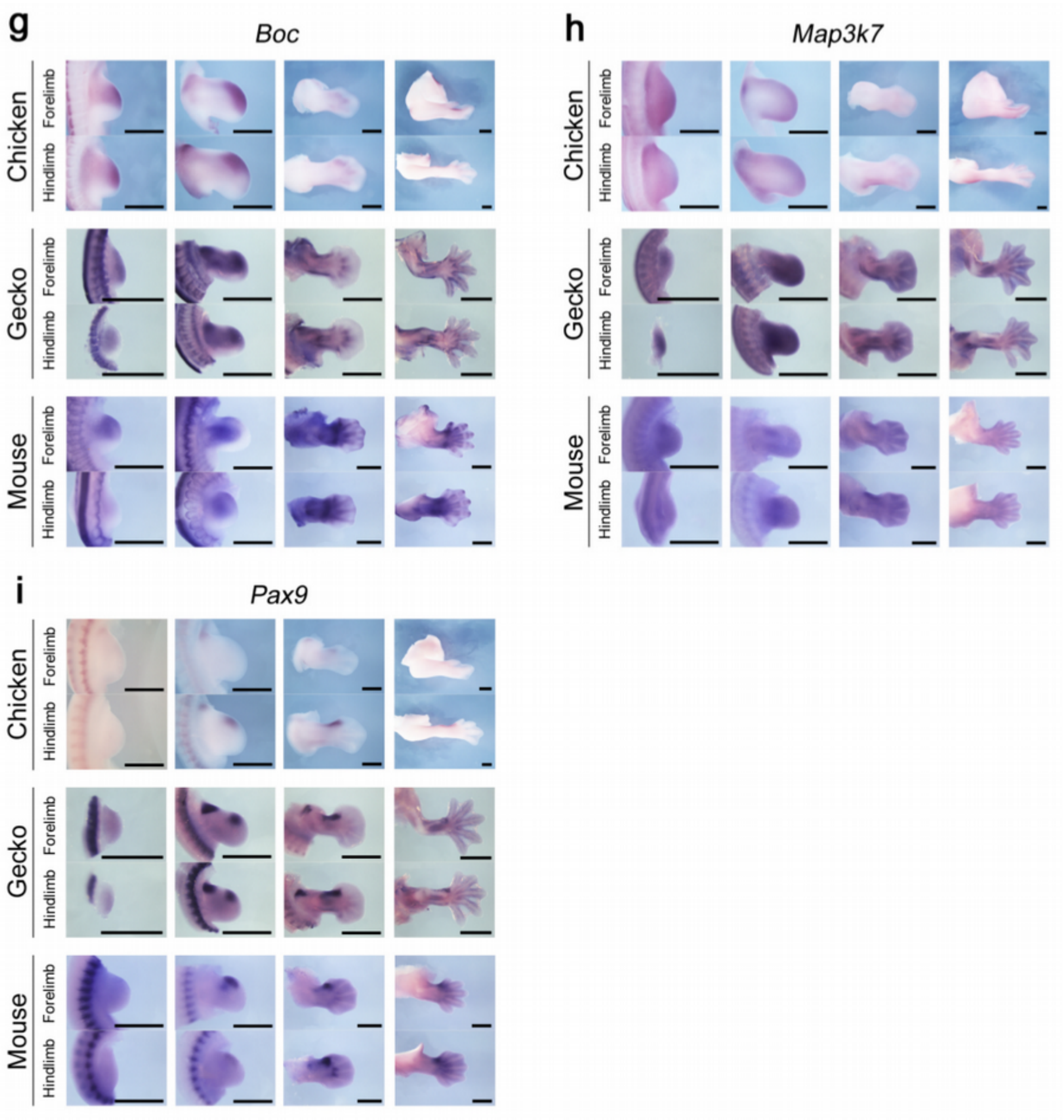

Supplementary Figure 6. ***in situ*** **hybridization assay of the second-screened genes in the gecko embryo.** A table showing expression profiles of 9 genes used for the final *in situ* expression screening with the developing gecko limb bud. Nine of 10 gecko orthologues of genes that were selected in the second screening (**Supplementary Fig. 5**) were successfully cloned from gecko embryonic cDNA. Gecko embryos at 6-7 dpo, 9-11 dpo, 16-18 dpo and 22-23 dpo, stages approximately corresponding to those of the other

embryos, were analyzed. To compare the expression patterns among three animals we used, the panels were arranged for each gene (**a**, *Inadl*; **b**, *Dach2*; **c**, *Rnf220*; **d**, *Pbx3*; **e**, *Sim1*; **f**, *Dpyd*; **g**, *Boc*; **h**, *Map3k7*; **i**, *Pax9*). *Inadl*, *Sim1*, *Boc* and *Pax9* were finally selected as candidates for genes that have avian-specific expression in the developing limb bud, because expression patterns of these genes in the chicken embryo were obviously different from those in gecko and mouse embryos. Some pictures are flip-flopped horizontally to arrange the proximo-distal and antero-posterior axes. See the main text and **Fig. 4** for details. Scale bars, 1 mm.

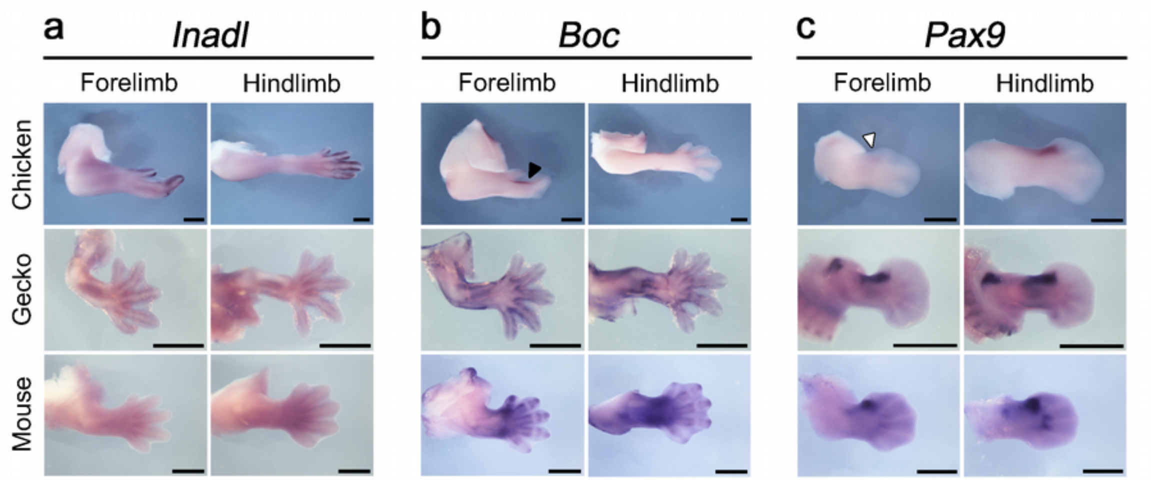

Supplementary Figure 7. Expression pattern of *Inadl*, *Boc*, *Pax9*, which exhibits specific expression profile in the developing chicken limb. (**a**) Expression pattern of *Inadl* in fore- and hindlimbs in chicken (HH33), gecko (23 dpo) and mouse (E13.5) embryos. (**b**) Expression pattern of *Boc* in fore- and hindlimbs in chicken (HH32), gecko (22 dpo) and mouse (E13.5) embryos. Black arrowhead indicates restricted expression in the anterior side of the second metacarpal in the chicken forelimb. (**c**) Expression pattern of *Pax9* in fore- and hindlimbs in chicken (HH29), gecko (16 dpo) and mouse (E12.5)

embryos. White arrowhead indicates apparently weak expression in the chicken forelimb. Scale bars, 1 mm.

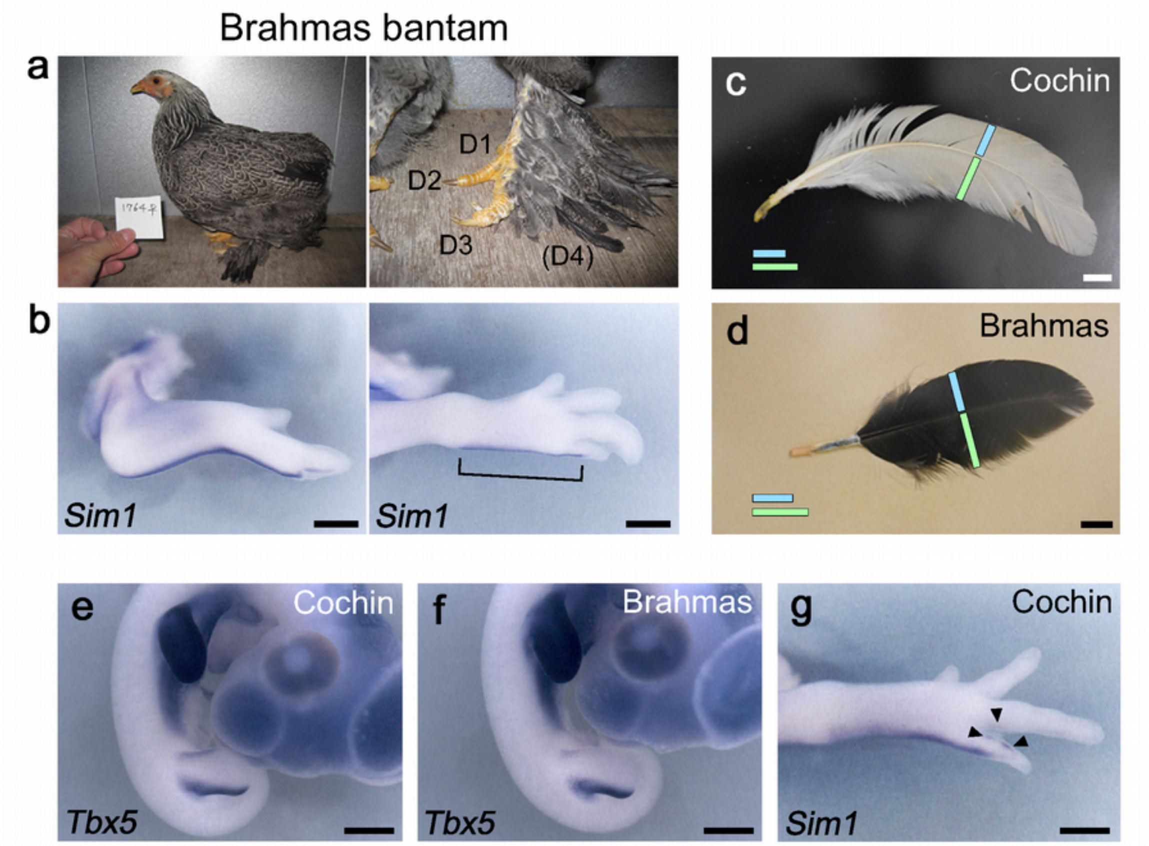

Supplementary Figure 8. Phenotypes and gene expression analysis of feathered feet chicken strains. (**a**) Photos of the whole body (left) and close-up of foot (right) of an adult Brahmas bantam. D1-4 incicate digits 1-4, respectively. Digit 4 is not seen from this angle because of heavily covering feathers. (**b**) Expression of Sim1 in the forelimb (left) and hindlimb (right) in the Brahmas bantam embryo at HH34. Bracket indicates *Sim1* expression in the hindlimb. (**c, d**) Feathers pulled out from the foot of the adult Cochin bantam (**c**) and Brahmas bantam (**d**). As blue and green bars indicate, these feathers show asymmetries of the vane width, a typical morphology of flight feathers. (**e**, **f**) Expression

of *Tbx5* in the Cochin bantam (**e**) and Brahmas bantam (**f**) embryos at HH25. Both embryos show strong and weak expression in the posterior and anterior-proximal parts of the hindlimb bud, respectively, in addition to the ubiquitous expression in the forelimb. *Tbx5* gene expression in the hindlimb, which previously identified to be linked with feathered feet phenotype in other chicken breeds and pigeons, suggests the same gene regulatory changes underlie generation of the feathered feet phenotypes of these breeds. (**g**) Expression of *Sim1* in the Cochin bantam embryo at HH36. Arrowheads indicate expression in the anterior side of digit 4 and posterior side of digit 3. Its expression at a later stage corresponds with the region where the feathery skin develops. Scale bars, 1 mm (**b**,**e-g**); 1 cm (**c**,**d**).

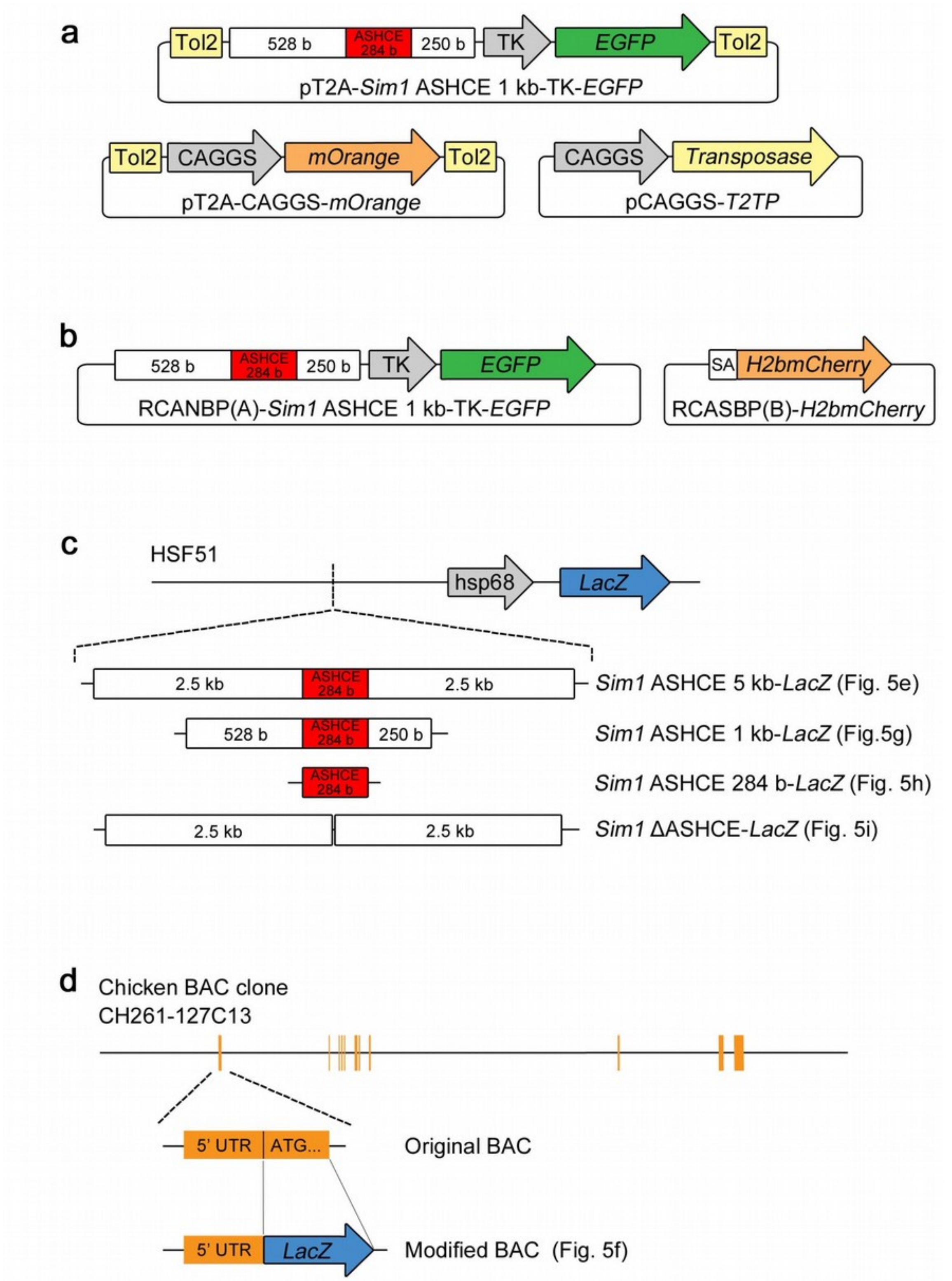

Supplementary Figure 9. Reporter vectors used for assays of a *Sim1*-associated ASHCE. (a, b) Images of reporter vectors used for assays in the chicken embryo by *in*

ovo electroporation (a) and retrovirus infection (b). (**c**) Images of reporter vectors used for generating transgenic mice. (**d**) Images of the chicken BAC clone (CH261-127C13) that contains *Sim1* locus and the modified (*LacZ*-inserted) BAC DNA. The original sequence from the first codon in the first exon to the exon end was replaced with a *LacZ* cassette. Orange boxes indicate the exons of *Sim1*. The *Sim1* ASHCE used for the reporter assay (in red) includes the highest-scoring ASHCE (284 b) in *Sim1* locus (see **Fig. 5a**).

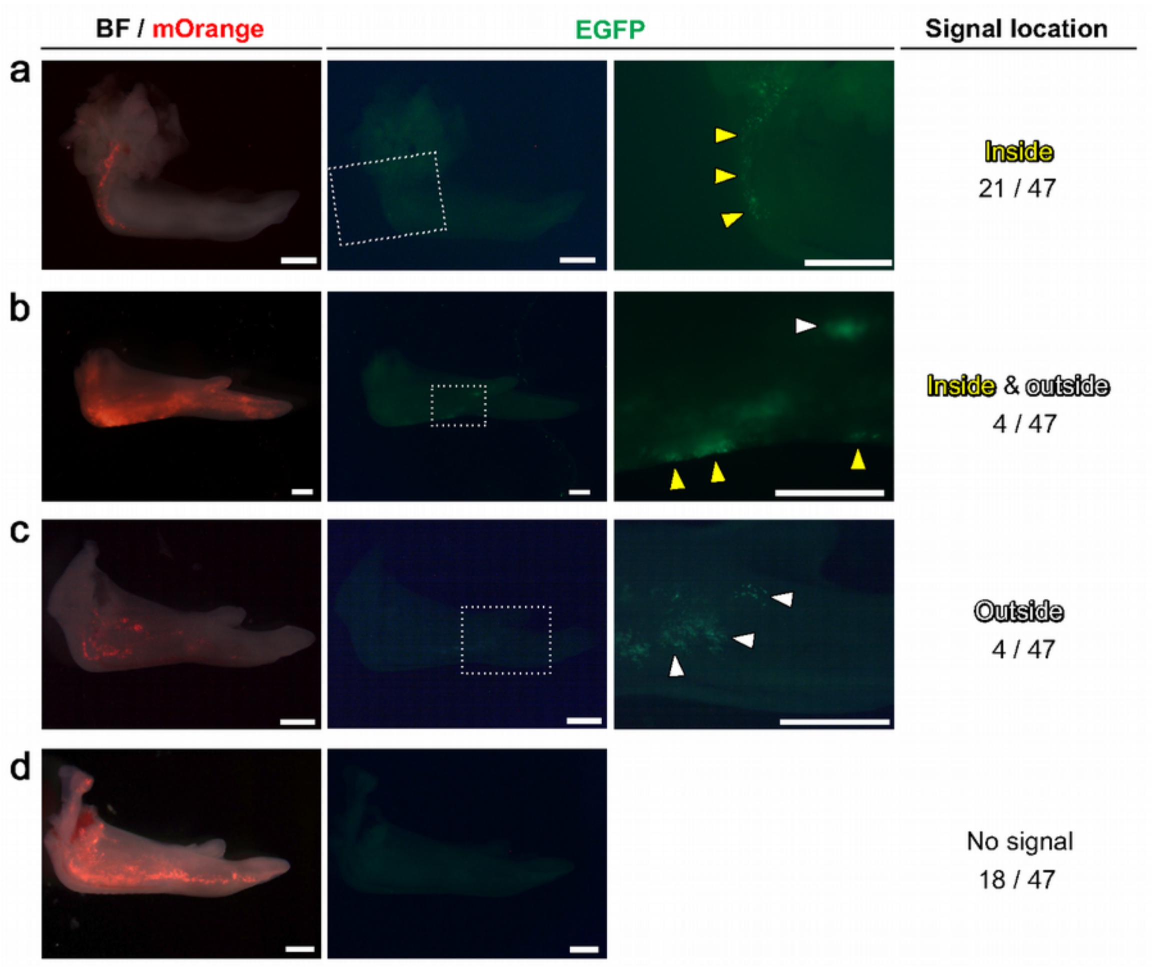

Supplementary Figure 10. Reporter expression of the *Sim1* ASHCE mainly overlapped with the endogenous expression of *Sim1* in chicken forelimb. Reporter activity of *Sim1* ASHCE 1 kb was examined by electroporation on chicken embryos (see **Fig. 5b, c** for details). Resultant embryos were classified into four categories according to the reporter signal location: inside (**a**), both inside and outside (**b**), outside (**c**) and no signal (**d**). Yellow arrowheads indicate the reporter expression inside/around endogenous expression domain of Sim1 in the posterior margin of chicken forelimb bud. White arrowheads indicate the reporter expression outside endogenous *Sim1* expression domain. The ratios in the right column mean the number of the embryos for each category to the

total number of embryos examined. Dotted boxes in the middle column are magnified to clearly show the EGFP signals in the left column. Scale bars, 1 mm.

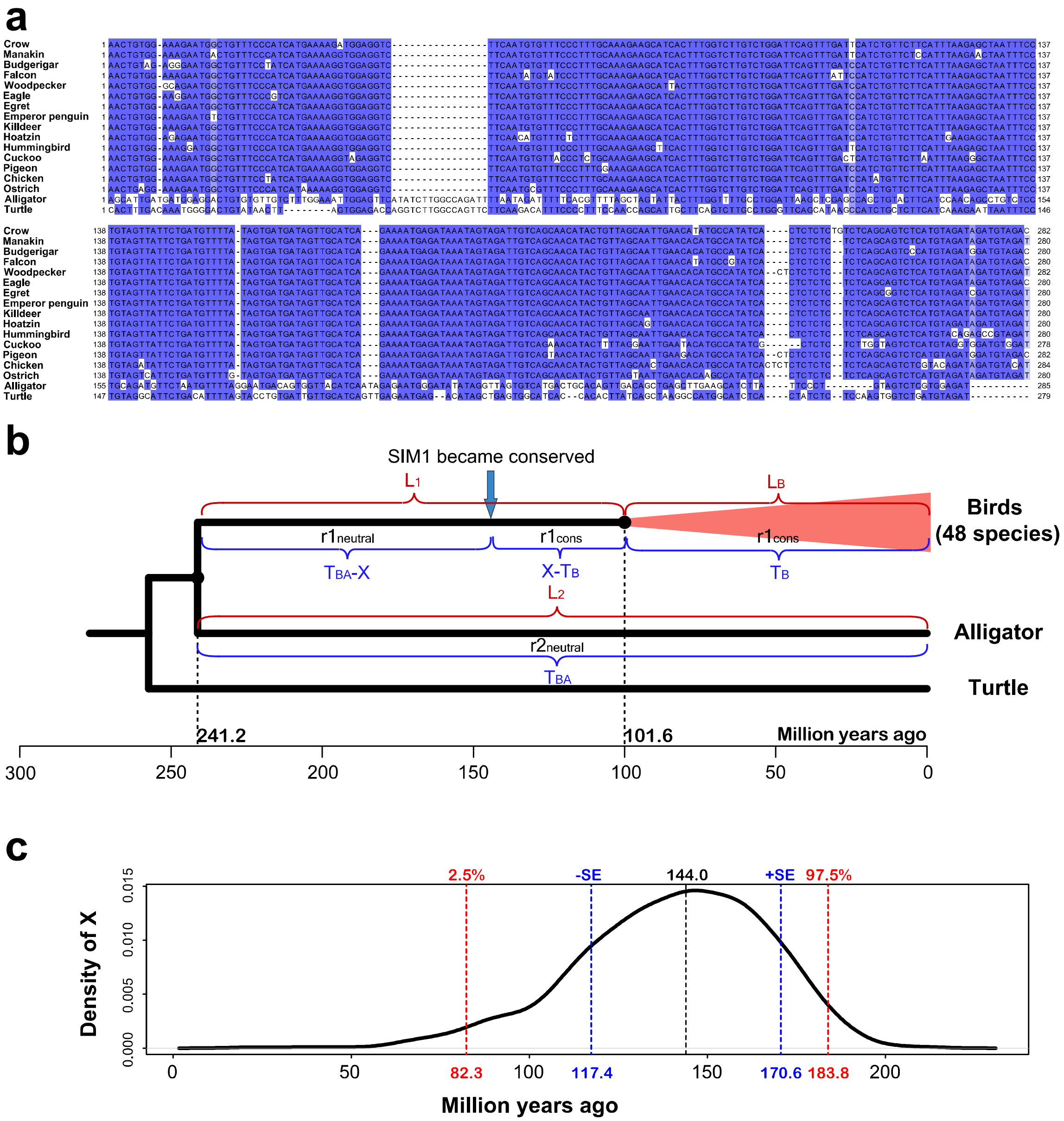
a
Crow
Manakin
Budgerigar
Falcon
Woodpecker
Eagle
Egret
Emperor penguin
Killdeer
Hoatzin
Hummingbird
Cuckoo
Pigeon
Chicken
Ostrich
Alligator
Turtle
b
SIM1 became conserved
L1
LB
L2
r1neutral
r1cons
r2neutral
TBA-X
X-TB
TB
TBA
Birds
(48 species)
Alligator
Turtle
241.2
101.6
Million years ago
300
250
200
150
100
50
0
c
Density of X
2.5%
-SE
144.0
+SE
97.5%
0.000
0.005
0.010
0.015
0
50
100
150
200
82.3
117.4
170.6
183.8
Million years ago

Supplementary Figure 11. Dating analysis of the *Sim1* ASHCE. (**a**) The alignments of 15 representative birds and two reptiles as outgroups in *Sim1* dating analysis. (**b**) Illustration of the method for estimating when the ASHCE element became conserved in birds. We assumed that since a specific time point (denoted by X Mya) in the common ancestor of all birds the element evolved at a conserved rate (denoted by $r1_{cons}$), and before that time point it evolved at a neutral rate $r1_{neutral}$. In addition, we assumed in the alligator lineage the element evolved at a neutral rate $r2_{neutral}$. We denoted the time of least common ancestor of 48 birds and alligator by T_{BA}, the time of least common ancestor of 48 birds by T_B, and the accumulative substitutions in birds from T_{BA} to T_B by L_1 (corresponding to the branch length in the phylogeny based on the alignments of this element). As $(T_{BA}-X)*r1_{neutral}+(X-T_B)*r1_{cons}=L_1$, thus we can get $X=(T_{BA}*r1_{neutral}-T_B*r1_{cons}-L_1)/(r1_{neutral}-r1_{cons})$. $r1_{cons}$ can be estimated by averaging the substitution rates of bird branches after T_B. $r1_{neutral}$ can be estimated based on the ratio of avian neutral substitution rate to alligator's neutral substitution rate. We denoted the neutral substitution rate in alligator's homolog of the *Sim1* ASHCE element by $r2_{neutral}$, the whole-genome neutral substitution rates of birds and alligators by $r1'_{neutral}$ and $r2'_{neutral}$ respectively. We assumed the ratio of avian neutral substitution rate to alligator's neutral substitution rate in the *Sim1* ASHCE is the same as that for whole genome, that is $r1_{neutral} : r2_{neutral} = r1'_{neutral} : r2'_{neutral}$, then we obtained $r1_{neutral}= (r1'_{neutral}/r2'_{neutral})/r2_{neutral}$. $r2_{neutral}$ was calculated by dividing the alligator branch length (L_2) on the phylogeny of the element by T_{BA}. Similarly, we calculated the $r1'_{neutral}$ and $r2'_{neutral}$ with phylogeny of the 4-fold degenerate sites (representing whole genome background) and divergence times (T_{BA} and T_B). The divergence times T_{BA} (241.2 Mya) and T_B (101.6 Mya) were obtained from previous

studies. (**c**) Estimating the statistical accuracy of X by bootstrapping. We generated 1000 bootstrap alignment replicates of the same length and inferred the phylogenies using phyloBoot in PHAST package[3]. According to the result of bootstrapping, the standard error of X is 26.6My and 95% confidence interval is (82.3, 183.8) Mya, respectively.

Supplementary Table 1. List of species used for gene family analysis and lineage-specific HCEs analysis."√" means the species was used in corresponding anlaysis.

Common name	Species name	Gene family	ASHCE	MSHCE
**Birds**				
Rifleman	*Acanthisitta chloris*		√	
Peking duck	*Anas platyrhynchos domestica*	√	√	
Chuk-will's-widow	*Antrostomus carolinensis*		√	
Bar-tailed trogon	*Apaloderma vittatum*		√	
Emperor penguin	*Aptenodytes forsteri*	√	√	
Grey-crowned crane	*Balearica regulorum gibbericeps*		√	
Rhinoceros hornbill	*Buceros rhinoceros silvestris*		√	
Anna's hummingbird	*Calypte anna*	√	√	
Cariama cristata	*Cariama cristata*		√	
Turkey vulture	*Cathartes aura*		√	
Chimney swift	*Chaetura pelagica*	√	√	
Killdeer	*Charadrius vociferus*	√	√	
MacQueen's bustard	*Chlamydotis macqueenii*		√	
Speckled mousebird	*Colius striatus*		√	
Pigeon	*Columba livia*	√	√	
American crow	*Corvus brachyrhynchos*	√	√	
Common cuckoo	*Cuculus canorus*	√	√	
Little egret	*Egretta garzetta*	√	√	
Sunbittern	*Eurypyga helias*		√	
Peregrine falcon	*Falco peregrinus*	√	√	
Northern fulmar	*Fulmarus glacialis*		√	
Chicken	*Gallus gallus*	√	√	√
Red-throated loon	*Gavia stellata*		√	
Medium ground-finch	*Geospiza fortis*	√	√	
White-tailed eagle	*Haliaeetus albicilla*		√	
Bald eagle	*Haliaeetus leucocephalus*	√	√	
Cuckoo roller	*Leptosomus discolor*		√	
Golden-collared manakin	*Manacus vitellinus*	√	√	
Turkey	*Meleagris gallopavo*	√	√	
Budgerigar	*Melopsittacus undulatus*	√	√	
Carmine bee-eater	*Merops nubicus*		√	
Brown mesite	*Mesitornis unicolor*		√	
Kea	*Nestor notabilis*		√	
Crested ibis	*Nipponia nippon*	√	√	
Hoatzin	*Opisthocomus hoazin*	√	√	
Dalmatian pelican	*Pelecanus crispus*		√	

White-tailed tropicbird	*Phaethon lepturus*		√	
Great cormorant	*Phalacrocorax carbo*		√	
American flamingo	*Phoenicopterus ruber ruber*		√	
Downy woodpecker	*Picoides pubescens*	√	√	
Great-crested grebe	*Podiceps cristatus*		√	
Yellow-throated sandgrouse	*Pterocles gutturalis*		√	
Penguin	*Pygoscelis adeliae*	√	√	
Common ostrich	*Struthio camelus australis*	√	√	
Zebra finch	*Taeniopygia guttata*	√	√	
Red-crested turaco	*Tauraco erythrolophus*		√	
White-throated tinamou	*Tinamus guttatus*		√	
Barn owl	*Tyto alba*		√	
**Mammals**				
Human	*Homo sapiens*	√	√	√
Chimpanzee	*Pan troglodytes*	√		√
Rhesus	*Macaca mulatta*	√		√
Bushbaby	*Otolemur garnetti*			√
Tree shrew	*Tupaia glis*	√		√
House mouse	*Mus musculus*	√	√	√
Brown rat	*Rattus norvegicus*	√	√	√
Guinea pig	*Cavia porcellus*			√
Rabbit	*Oryctolagus cuniculus*	√		√
Shrew	*Sorex araneus*			√
Hedgehog	*Erinaceus europaeus*			√
Dog	*Canis familiaris*	√		√
Cat	*Felis catus*	√		√
Horse	*Equus caballus*	√		√
Cow	*Bos taurus*	√		√
Armadillo	*Dasypus novemcinctus*			√
Elephant	*Loxodonta africana*	√		√
Tenrec	*Echinops telfairi*			√
Gray short-tailed opossum	*Monodelphis domestica*	√	√	√
Platypus	*Ornithorhynchus anatinus*	√		√
Panda	*Ailuropoda melanoleuca*	√		
Marmoset	*Callithrix jacchus*	√		
Gorilla	*Gorilla gorilla*	√		
Naked mole-rat	*Heterocephalus glaber*	√		
Squirrel	*Ictidomys tridecemlineatus*	√		
Ferret	*Mustela putorius furo*	√		
Myotis	*Myotis davidii*	√		
Orangutan	*Pongo abelii*	√		
Fruit bat	*Pteropus alecto*	√		

Boar	*Sus scrofa*	√		
**Other Reptiles**				
Green anole lizard	*Anolis carolinensis*	√	√	√
Snake	*Boa constrictor*	√		
Softshell turtle	*Pelodiscus sinensis*	√		
American alligator	*Alligator mississippi*	√	√	
Green sea turtle	*Chelonia mydas*	√	√	
**Fishes**				
Fugu	*Takifugu rubripes*	√		√
Tetraodon	*Tetraodon nigroviridis*	√		√
Stickleback	*Gasterosteus aculeatus*	√		√
Medaka	*Oryzias latipes*	√		√
Zebrafish	*Danio rerio*	√	√	√
Bowfin	*Amia calva*	√		
Boleophthalmus	*Boleophthalmus pectinirostris*	√		
Cod	*Gadus morhua*	√		
Gar	*Lepisosteus oculatus*	√		
Periophthalmus	*Periophthalmus magnuspinnatus*	√		
Platyfish	*Xiphophorus maculatus*	√		
**Others**				
Western clawed frog	*Xenopus tropicalis*		√	√

Supplementary Table 2. The statistics of multiple alignments of 48 birds plus 9 outgroups. The names in brackets indicate the UCSC assembly versions.

English species common name	aligned length(Mb)	%genome
**Birds**		
Rifleman	388.84	37.03%
Pekin duck	401.46	36.50%
bar-tailed trogon	390.46	36.15%
emperor penguin	395.90	31.42%
grey-crowned crane	395.29	34.67%
rhinoceros hornbill	390.42	36.15%
Anna's hummingbird	372.98	33.91%
chuck-will's-widow	394.98	34.35%
red-legged seriema	392.05	34.09%
turkey vulture	393.59	33.64%
chimney swift	378.91	34.45%
Killdeer	393.03	32.75%
MacQueen's bustard	388.10	35.61%
Pigeon	393.18	35.42%

speckled mousebird	376.23	34.84%
American crow	387.98	35.27%
common cuckoo	391.70	34.06%
little egret	391.34	32.61%
Sunbittern	383.40	34.85%
peregrine falcon	394.01	33.39%
northern fulmar	395.72	34.71%
Chicken	394.55	37.58%
red-throated loon	395.84	34.42%
medium ground-finch	382.88	35.78%
white-tailed eagle	393.12	34.48%
cuckoo-roller	393.91	34.25%
golden-collared manakin	388.83	34.72%
Turkey	392.94	35.72%
Budgerigar	382.04	34.73%
carmine bee-eater	379.99	35.85%
brown mesite	382.50	34.77%
Kea	386.38	33.89%
crested ibis	396.07	33.85%
Hoatzin	388.31	34.06%
dalmatian pelican	393.54	33.64%
great cormorant	394.88	34.34%
white-tailed tropicbird	390.66	33.68%
American flamingo	394.27	34.58%
downy woodpecker	347.21	29.68%
great-crested grebe	389.67	33.88%
yellow-throated sandgrouse	386.40	36.11%
Adelie penguin	395.24	32.13%
common ostrich	398.75	32.42%
zebra finch	321.74	26.81%
red-crested turaco	389.99	33.33%
white-throated tinamou	362.50	34.52%
barn owl	394.96	34.65%
**Reptiles**		
American alligator	287.63	13.24%
green sea turtle	339.60	15.19%
green anole lizard (anocar1)	110.15	6.18%
**Mammals**		
human (hg18)	83.51	2.67%
house mouse (mm8)	63.39	2.35%

brown rat (rn4)	60.02	2.14%
gray short-tailed opossum (mondom4)	83.90	2.33%
**Amphibian**		
western clawed frog (xentro2)	49.28	3.29%
**Fish**		
zebrafish (danrer4)	42.48	3.03%

Supplementary Table 3. Statistics of all avian HCEs and ASHCEs.

	**all avian HCEs**		**ASHCEs**	
	**#segments**	**Total length (bp)**	**#segments**	**Total length (bp)**
**>=20bp**	1,441,723	66,243,831	265984	10,965,592
**>=100bp**	100,190	16,656,033	13,354	1,895,050
**>=200bp**	20,376	6,053,112	1376	333,817
**>=500bp**	1,225	777,262	0	0
**>=1000bp**	32	38,003	0	0

Supplementary Table 4. Statistics of all mammalian HCEs and MSHCEs.

	**All HCEs**		**MSHCEs**	
	**#segments**	**Total length (bp)**	**#segments**	**Total length (bp)**
**>=20bp**	1074348	78,420,350	283,323	20,366,803
**>=100bp**	222190	41,415,022	55,436	10,756,896
**>=200bp**	59064	19,062,100	17,557	5,507,870
**>=500bp**	5784	4,089,668	1,382	962,099
**>=1000bp**	567	725,492	128	161,100

Supplementary Table 5. Classification of ASHCEs and MSHCEs.

		**Coding**	**5' 10kb**	**3' 10kb**	**Intron**	**Intergenic**	**Total**
**ASHCE**	**#bases**	33,462	889,880	790,696	3,011,221	6,240,333	10,965,592
	**%**	0.31	8.12	7.21	27.46	56.91	100

**MSHCE**	**#bases**	835,958	1,657,564	1,664,665	3,811,455	12,397,161	20,366,803
	**%**	4.10	8.14	8.17	18.71	60.87	100

Supplementary Table 6. Homologs of ASHCEs in other outgroups. 'All vertebrates' means the ASHCE homolog exists in at least one species for each of the 4 groups (reptile, mammal, amphibian and fish). See Supplementary Table 1 **for details of species in each group.**

**Outgroups**	**Length found in outgroups**	**Length % of ASHCEs**	**Mean length (bp)**	**# of homologs found in outgroups**	**Number % of ASHCEs**
At least one outgroup	4,857,546	43.25%	54.36	89,355	33.59%
Reptiles	4,846,867	43.15%	54.74	88,549	33.39%
Mammals	552,771	4.92%	44.73	12,358	4.65%
Amphibian	57,104	0.51%	35.51	1,608	0.6%
Fish	28,337	0.25%	30.67	924	0.35%
All vertebrates	9,719	0.087%	29.45	330	0.12%

Supplementary Table 7. Substitution rates based on the alignments of the ASHCE loci with at least one outgroup. **The substitution rates are in units of substitutions per site per million years.**

Species	Substitution rate	Mean rate	Median rate
**Birds**			
zebra finch	0.000681543	0.000431762	0.000403021
medium ground-finch	0.000659996	0.000431762	0.000403021
American crow	0.000587916	0.000431762	0.000403021
golden-collared manakin	0.000539072	0.000431762	0.000403021
rifleman	0.000523038	0.000431762	0.000403021
kea	0.000466174	0.000431762	0.000403021
budgerigar	0.000534012	0.000431762	0.000403021
speckled mousebird	0.000508680	0.000431762	0.000403021
carmine bee-eater	0.000486299	0.000431762	0.000403021
downy woodpecker	0.000690200	0.000431762	0.000403021
rhinoceros hornbill	0.000524563	0.000431762	0.000403021
bar-tailed trogon	0.000463027	0.000431762	0.000403021
cuckoo-roller	0.000357719	0.000431762	0.000403021
peregrine falcon	0.000371959	0.000431762	0.000403021
red-legged seriema	0.000341981	0.000431762	0.000403021
white-tailed eagle	0.000295532	0.000431762	0.000403021
bald eagle	0.000293547	0.000431762	0.000403021
turkey vulture	0.000278690	0.000431762	0.000403021
barn owl	0.000346620	0.000431762	0.000403021
crested ibis	0.000299974	0.000431762	0.000403021
little egret	0.000337963	0.000431762	0.000403021
great cormorant	0.000370965	0.000431762	0.000403021
dalmatian pelican	0.000314179	0.000431762	0.000403021
Adelie penguin	0.000304496	0.000431762	0.000403021
emperor penguin	0.000297309	0.000431762	0.000403021
northern fulmar	0.000299974	0.000431762	0.000403021
red-throated loon	0.000301863	0.000431762	0.000403021
sunbittern	0.000415654	0.000431762	0.000403021
white-tailed tropicbird	0.000351314	0.000431762	0.000403021
great-crested grebe	0.000353308	0.000431762	0.000403021
American flamingo	0.000281589	0.000431762	0.000403021
killdeer	0.000307310	0.000431762	0.000403021
grey-crowned crane	0.000306545	0.000431762	0.000403021
MacQueen's bustard	0.000405714	0.000431762	0.000403021
red-crested turaco	0.000368710	0.000431762	0.000403021
hoatzin	0.000400327	0.000431762	0.000403021
common cuckoo	0.000511454	0.000431762	0.000403021
pigeon	0.000472850	0.000431762	0.000403021
yellow-throated sandgrouse	0.000395502	0.000431762	0.000403021
brown mesite	0.000436203	0.000431762	0.000403021
Anna's hummingbird	0.000567773	0.000431762	0.000403021
chimney swift	0.000498870	0.000431762	0.000403021

chuck-will's-widow	0.000355176	0.000431762	0.000403021
chicken	0.000606479	0.000431762	0.000403021
turkey	0.000643255	0.000431762	0.000403021
pekin duck	0.000426759	0.000431762	0.000403021
white-throated tinamou	0.000717969	0.000431762	0.000403021
common ostrich	0.000424512	0.000431762	0.000403021
**Reptiles**			
American alligator	0.001140133	0.001159919	0.001140133
green sea turtle	0.000544678	0.001159919	0.001140133
green anole lizard	0.001794946	0.001159919	0.001140133
**Mammals**			
human	0.001355605	0.001479022	0.001496517
brown rat	0.001658961	0.001479022	0.001496517
house mouse	0.001637429	0.001479022	0.001496517
gray short-tailed opossum	0.001264091	0.001479022	0.001496517
**Amphibian**			
western clawed frog	0.001903663	0.001903663	0.001903663

Supplementary Table 8. Statistics of chicken SNPs in HCEs.

	**#SNP**	**Total length(bp)**	**#SNP per kb**	**Chi-squared test (with genome background)**	**Chi-square test (with coding region)**
All HCEs	109,828	111,928,291	0.98	p < 2.2e-16	p < 2.2e-16
ASHCEs	30,254	23,660,241	1.27	p < 2.2e-16	p=0.1727
Coding region	31,478	24,343,227	1.29	p < 2.2e-16	-
whole chicken genome	2,871,325	1,105,595,305	2.59	-	-

Supplementary Table 9. Over-represented TFBSs (including ChIP-seq motifs predicted by Homer) in ASHCEs. *GAT* was used to identify the over-represented TFBS and ChIP-seq motifs compared to the whole genome background (q-value<0.05). Motif names with ”*” indicate corresponding transcription factors that are involved in regulation of developmental process (GO:0050793) based on the chicken ENSEMBL GO annotation.

Motif matrix	TF name	Observed length(bp)	Expected length(bp)	Fold	q-value
De-novo-TAATTAGC	BestGuess:Lhx2(Homeobox)	13,094	4,453	2.9	0.001
homer_known_1	RFX(HTH)	9,791	3,564	2.7	0.001
homer_known_38	OCT:OCT(POU,Homeobox,IR1)	5,673	2,136	2.7	0.001
homer_known_20	Rfx2(HTH)	10,995	4,258	2.6	0.001
MA0091.1	TAL1::TCF3	26,493	10,313	2.6	0.001
homer_known_5	Pax7(Paired,Homeobox),long	6,004	2,521	2.4	0.001
MA0052.1	MEF2A	16,125	7,090	2.3	0.001
MA0142.1	Pou5f1	1,484	658	2.3	0.001
MA0125.1	Nobox	84,192	37,893	2.2	0.001
homer_known_44	TCFL2(HMG)	9,940	4,476	2.2	0.001
homer_known_22	Mef2d(MADS)	33,685	15,208	2.2	0.001
MA0135.1	Lhx3	13,789	6,347	2.2	0.001
homer_known_42	Nrf2(bZIP)	4,472	2,080	2.1	0.001
MA0132.1	Pdx1	79,778	37,935	2.1	0.001
MA0143.1*	Sox2	1,284	613	2.1	0.001
MA0063.1*	Nkx2-5	31,216	14,990	2.1	0.001
homer_known_60*	NF1:FOXA1(CTF,Forkhead)	11,229	5,419	2.1	0.001
homer_known_51	Pax7(Paired,Homeobox),longest	8,003	3,951	2.0	0.001
MA0046.1	HNF1A	2,520	1,244	2.0	0.001
homer_known_27	Pax7(Paired,Homeobox)	14,718	7,311	2.0	0.001
homer_known_8	X-box(HTH)	13,442	6,721	2.0	0.001
homer_known_17	Nur77(NR)	22,965	11,681	2.0	0.001
homer_known_58	NFkB-p65-Rel(RHD)	4,199	2,150	2.0	0.001
homer_known_19	Tcf3(HMG)	29,810	15,279	2.0	0.001
homer_known_39	OCT:OCT(POU,Homeobox)	1,519	780	1.9	0.001
homer_known_45	Hoxb4(Homeobox)	26,878	13,926	1.9	0.001
MA0099.2	AP1	46,739	25,020	1.9	0.001
MA0159.1	RXR::RAR_DR5	270	145	1.9	0.007
homer_known_40*	HOXA2(Homeobox)	12,091	6,788	1.8	0.001
MA0071.1	RORA_1	42,543	23,945	1.8	0.001
homer_known_3	Hnf1(Homeobox)	22,965	13,017	1.8	0.001
MA0153.1*	HNF1B	6,640	3,769	1.8	0.001
MA0158.1	HOXA5	48,526	28,141	1.7	0.001
MA0017.1	NR2F1	2,353	1,371	1.7	0.001
MA0151.1	ARID3A	144,859	86,098	1.7	0.001
homer_known_30	OCT4-SOX2-TCF-NANOG(POU,Homeobox,HMG)	37,799	22,907	1.7	0.001
homer_known_21	ETS:E-box(ETS,bHLH)	7,558	4,609	1.6	0.001
homer_known_13	Ets1-distal(ETS)	19,040	11,624	1.6	0.001
homer_known_48*	PAX6(Paired,Homeobox)	14,713	9,069	1.6	0.001
MA0083.1*	SRF	225	138	1.6	0.023

MA0047.2	Foxa2	32,740	20,497	1.6	0.001
MA0144.1*	Stat3	17,057	10,715	1.6	0.001
homer_known_55	TR4(NR),DR1	4,684	2,982	1.6	0.001
MA0137.2*	STAT1	5,636	3,591	1.6	0.001
MA0029.1	Evi1	1,267	811	1.6	0.001
MA0114.1*	HNF4A	9,952	6,561	1.5	0.001
homer_known_28	LXRE(NR),DR4	3,763	2,485	1.5	0.001
MA0070.1*	PBX1	5,413	3,600	1.5	0.001
MA0141.1*	Esrrb	16,304	10,899	1.5	0.001
homer_known_54*	FOXP1(Forkhead)	51,460	34,663	1.5	0.001
homer_known_2	GATA:SCL(Zf,bHLH)	18,540	12,596	1.5	0.001
MA0009.1	T	1,231	839	1.5	0.001
MA0030.1	FOXF2	7,333	5,010	1.5	0.001
MA0092.1	Hand1::Tcfe2a	71,366	48,867	1.5	0.001
MA0065.2*	PPARG::RXRA	1,061	732	1.4	0.001
MA0090.1	TEAD1	9,488	6,624	1.4	0.001
homer_known_12	TEAD2(TEA)	33,435	23,479	1.4	0.001
homer_known_46	Rfx5(HTH)	21,213	14,936	1.4	0.001
MA0150.1*	NFE2L2	13,748	9,727	1.4	0.001
homer_known_33	Six1(Homeobox)	20,618	14,655	1.4	0.001
De-novo-AAATAACAGCGC	BestGuess:Rhox11	8,590	6,148	1.4	0.001
homer_known_24	NFAT:AP1(RHD,bZIP)	30,228	22,055	1.4	0.001
MA0136.1*	ELF5	78,752	57,912	1.4	0.001
MA0157.1*	FOXO3	95,727	70,787	1.4	0.001
MA0164.1	Nr2e3	45,023	33,362	1.3	0.001
MA0077.1*	SOX9	48,046	35,608	1.3	0.001
MA0162.1	Egr1	2,759	2,061	1.3	0.001
MA0102.2	CEBPA	36,694	27,787	1.3	0.001
MA0043.1	HLF	12,702	9,707	1.3	0.001
MA0087.1	Sox5	74,668	57,161	1.3	0.001
MA0108.2	TBP	29,585	22,669	1.3	0.001
MA0160.1	NR4A2	37,270	28,624	1.3	0.001
MA0038.1	Gfi	98,206	75,566	1.3	0.001
De-novo-TTTAAMCGCG	BestGuess:MBP1::SWI6	7,120	5,544	1.3	0.001
MA0080.2*	SPI1	19,902	15,531	1.3	0.001
homer_known_36	TATA-box	6,688	5,271	1.3	0.001
homer_known_57	RAR:RXR(NR),DR5	1,462	1,156	1.3	0.013
homer_known_34	PU.1:IRF8(ETS:IRF)	15,161	11,993	1.3	0.001
MA0048.1	NHLH1	7,558	6,015	1.3	0.001
homer_known_6	Oct4:Sox17(POU,Homeobox,HMG)	19,351	15,593	1.2	0.001
MA0101.1	REL	26,122	21,058	1.2	0.001
MA0025.1	NFIL3	14,635	11,815	1.2	0.001

homer_known_4	IRF2(IRF)	7,575	6,142	1.2	0.001
homer_known_56	ZNF317(Zf)	7,642	6,294	1.2	0.001
MA0018.2*	CREB1	8,626	7,201	1.2	0.001
homer_known_47	ETS(ETS)	7,427	6,214	1.2	0.001
MA0145.1	Tcfcp2l1	3,833	3,238	1.2	0.004
homer_known_35	IRF:BATF(IRF:bZIP)	15,020	12,850	1.2	0.001
MA0002.2*	RUNX1	52,294	45,015	1.2	0.001
De-novo-AAACAAACGCCG	BestGuess:Sox3	8,710	7,550	1.2	0.001
homer_known_26*	IRF1(IRF)	10,387	9,045	1.1	0.001
homer_known_59	Dorsal(RHD)	10,068	8,788	1.1	0.001
homer_known_29	ISRE(IRF)	3,939	3,475	1.1	0.008
De-novo-CGGBWWTN	BestGuess:YRR1	5,728	5,087	1.1	0.001
De-novo-GCGGTTTGCTTB	BestGuess:prd	5,824	5,236	1.1	0.005
MA0152.1*	NFATC2	93,563	84,557	1.1	0.001
MA0042.1	FOXI1	86,550	79,774	1.1	0.001
MA0100.1	Myb	13,776	12,961	1.1	0.005

Supplementary Table 10. Secondary RNA structures in ASHCEs predicted by Evofold. When counting the numbers of RNA structures overlapping expressed transcripts, Ensembl ncRNAs and ASHCEs, we required a minimum overlapping coverage of 50%.

**Length (bp)**	**#RNA structure**	**Expressed number**	**Expressed (%)**	**#overlap with Ensembl ncRNA**	**#overlap with ASHCEs**	**#overlap with ASHCEs and expressed**
>1	53225	40201	75.5	103	5511	919
>=20	29428	22071	75	81	2311	360
>=50	13995	10299	73.6	59	654	87
>=100	6063	4394	72.5	5	170	19
>=200	1405	924	65.8	0	16	1

Supplementary Table 11. microRNAs overlapping with ASHCEs. **A minimum overlapping coverage of 50% was used.**

**microRNA ID**	**% of length covered by ASHCEs**
MIMAT0007496	0.5454546

MIMAT0007547	0.9523810
MIMAT0007599	0.8695652
MIMAT0007490	1.0000000
MIMAT0007345	0.8695652
MIMAT0007346	1.0000000
MIMAT0007725	1.0000000
MIMAT0007696	1.0000000
MIMAT0007324	1.0000000
MIMAT0007560	0.9090909
MIMAT0007408	1.0000000
MIMAT0007663	1.0000000
MIMAT0011207	1.0000000

Supplementary Table 12. General statistics of ChIP-seq data. Each replicates was marked by r1 or r2. Used mapped Reads were uniquely mapped, after removing PCR duplicates and singletons. The normalized strand coefficient (NSC) and the relative strand correlation (RSC) were calculated by *SPP*.

Sample_name	Total Reads	%GC	%Q20	%Q30	Mapped reads	Mapped rate(%)	Used mapped Reads	Used mapped rate(%)	NSC	RSC
HH16_whole_H3K27ac_r1	29,451,107	45.94	99.05	97.20	27,167,485	92.25	24,491,314	83.16	1.033	1.009
HH16_whole_H3K27ac r2	42,038,672	46.43	98.80	96.41	37,836,106	90.00	31,241,879	74.32	1.134	1.496
HH16_whole_H3K27me3_r1	24,683,855	55.37	97.64	94.39	20,054,715	81.25	15,579,253	63.12	1.610	1.190
HH16_whole_H3K27me3_r2	28,843,698	46.46	99.01	97.08	26,061,026	90.35	22,712,883	78.74	1.036	0.700
HH16_whole_H3K4me1_r1	51,645,301	43.24	96.25	91.05	48,172,887	93.28	44,848,225	86.84	1.030	1.700
HH16_whole_H3K4me1_r2	32,069,427	45.68	96.93	93.44	29,220,234	91.12	26,796,051	83.56	1.050	1.380
HH21_limb_H3K27ac_r1	38,282,283	48.77	98.72	96.17	34,146,718	89.20	37,582,897	98.17	1.108	1.502
HH21_limb_H3K27ac r2	45,590,078	47.75	98.73	96.24	40,919,912	89.76	33,506,375	73.49	1.038	1.157
HH21_limb_H3K27me3_r1	30,330,674	54.69	98.12	95.08	25,558,154	84.27	20,579,352	67.85	1.450	1.060
HH21_limb_H3K27me3 r2	10,252,469	55.12	98.33	96.54	8,457,162	82.49	6,456,091	62.97	1.750	1.280
HH21_limb_H3K4me1_r1	24,133,423	49.17	97.56	93.51	22,057,298	91.40	19,600,485	81.22	1.070	1.020
HH21_limb_H3K4me1_r2	22,313,821	44.42	96.97	94.24	20,443,844	91.62	18,671,310	83.68	1.050	1.150
HH21_whole_H3K27ac_r1	27,875,287	44.61	99.02	97.14	25,887,392	92.87	23,673,431	84.93	1.048	1.937
HH21_whole_H3K27ac_r2	40,066,233	45.80	98.90	96.58	36,719,361	91.65	34,805,434	86.87	1.064	1.447
HH21_whole_H3K27me3_r1	17,796,085	49.84	97.57	93.51	15,218,878	85.52	12,209,388	68.61	1.340	0.910
HH21_whole_H3K27me3 r2	32,695,064	47.56	98.45	95.95	29,542,563	90.36	25,693,889	78.59	1.028	0.723
HH21_whole_H3K4me1_r1	24,231,184	48.61	97.26	92.94	22,168,518	91.49	19,738,328	81.46	1.070	1.050
HH21_whole_H3K4me1_r2	25,284,961	47.22	98.77	96.84	23,469,964	92.82	20,999,806	83.05	1.050	0.900
HH32_limb_H3K27ac_r1	42,481,863	46.06	98.73	96.40	38,869,950	91.50	35,168,260	82.78	1.042	1.156
HH32_limb_H3K27ac_r2	27,822,785	43.94	99.10	97.66	25,980,925	93.38	23,847,467	85.71	1.046	1.474
HH32_limb_H3K27me3_r1	27,776,109	51.06	98.88	96.92	24,317,387	87.55	20,616,744	74.22	1.220	1.040
HH32_limb_H3K27me3_r2	38,921,160	48.45	98.50	95.88	34,655,198	89.04	30,272,091	77.78	1.051	0.953
HH32_limb_H3K4me1_r1	49,133,949	42.16	96.22	91.48	45,390,661	92.38	42,409,219	86.31	1.030	1.490

HH32_limb_H3K4me1_r2	35,431,383	50.49	98.59	96.34	32,094,524	90.58	28,646,579	80.85	1.080	1.310
HH32_whole_H3K27ac_r1	27,174,933	54.25	97.83	94.66	23,076,431	84.92	20,377,315	74.99	1.420	1.250
HH32_whole_H3K27ac_r2	29,444,667	45.24	98.98	97.02	27,184,135	92.32	24,657,869	83.74	1.047	1.294
HH32_whole_H3K27me3_r1	40,928,441	50.33	96.23	91.70	34,674,192	84.72	30,791,000	75.23	1.340	1.240
HH32_whole_H3K27me3_r2	29,494,158	46.90	98.91	96.81	26,807,140	90.89	23,506,804	79.70	1.035	0.787
HH32_whole_H3K4me1_r1	25,141,278	46.51	96.76	91.94	23,106,685	91.91	21,038,184	83.68	1.050	1.140
HH32_whole_H3K4me1_r2	47,868,159	43.75	97.29	93.90	44,252,989	92.45	41,058,588	85.77	1.030	1.490

Supplementary Table 13. Statistics of peak calling.

Sample	Peak Number	Total Length	Average Length	Genome Rate(%)
HH16_whole_H3K27ac_r1	38,302	53,166,911	1,388	4.8
HH16_whole_H3K27ac_r2	52,550	88,216,830	1,678	7.96
HH16_whole_H3K27me3_r1	25,218	42,401,202	1,681	3.83
HH16_whole_H3K27me3_r2	13,592	20,972,975	1,543	1.89
HH16_whole_H3K4me1_r1	71,824	98,634,975	1,373	8.9
HH16_whole_H3K4me1_r2	58,161	75,438,304	1,297	6.81
HH21_limb_H3K27ac_r1	40,063	66,981,313	1,671	6.04
HH21_limb_H3K27ac_r2	41,285	65,812,613	1,594	5.94
HH21_limb_H3K27me3_r1	22,702	46,462,854	2,046	4.19
HH21_limb_H3K27me3_r2	14,811	29,009,907	1,958	2.62
HH21_limb_H3K4me1_r1	68,031	91,020,298	1,337	8.21
HH21_limb_H3K4me1_r2	39,238	41,300,663	1,052	3.73
HH21_whole_H3K27ac_r1	43,874	67,769,948	1,544	6.11
HH21_whole_H3K27ac_r2	36,474	49,182,273	1,348	4.44
HH21_whole_H3K27me3_r1	22,367	39,238,819	1,754	3.54
HH21_whole_H3K27me3_r2	53,164	60,608,006	1,140	5.47
HH21_whole_H3K4me1_r1	81,494	114,154,393	1,400	10.3
HH21_whole_H3K4me1_r2	77,330	94,633,527	1,223	8.54
HH32_limb_H3K27ac_r1	47,986	72,259,799	1,505	6.52
HH32_limb_H3K27ac_r2	45,583	66,797,500	1,465	6.03
HH32_limb_H3K27me3_r1	32,674	58,824,972	1,800	5.31
HH32_limb_H3K27me3_r2	37,031	61,755,598	1,667	5.57
HH32_limb_H3K4me1_r1	64,481	76,958,471	1,193	6.94
HH32_limb_H3K4me1_r2	87,525	111,903,327	1,278	10.1
HH32_whole_H3K27ac_r1	49,077	77,578,243	1,580	7
HH32_whole_H3K27ac_r2	40,600	60,456,378	1,489	5.45
HH32_whole_H3K27me3_r1	18,334	36,518,305	1,991	3.29

HH32_whole_H3K27me3_r2	23,545	39,447,494	1,675	3.56
HH32_whole_H3K4me1_r1	53,971	72,667,811	1,346	6.56
HH32_whole_H3K4me1_r2	45,393	50,514,676	1,112	4.56

Supplementary Table 14. The Pearson correlations between peaks of replicates.

Replicate A	Replicate B	Correlation
HH16_whole_H3K27ac_r1	HH16_whole_H3K27ac_r2	0.801
HH16_whole_H3K27me3_r1	HH16_whole_H3K27me3_r2	0.774
HH16_whole_H3K4me1_r1	HH16_whole_H3K4me1_r2	0.695
HH21_limb_H3K27ac_r1	HH21_limb_H3K27ac_r2	0.924
HH21_limb_H3K27me3_r1	HH21_limb_H3K27me3_r2	0.902
HH21_limb_H3K4me1_r1	HH21_limb_H3K4me1_r2	0.441
HH21_whole_H3K27ac_r1	HH21_whole_H3K27ac_r2	0.748
HH21_whole_H3K27me3_r1	HH21_whole_H3K27me3_r2	0.504
HH21_whole_H3K4me1_r1	HH21_whole_H3K4me1_r2	0.686
HH32_limb_H3K27ac_r1	HH32_limb_H3K27ac_r2	0.685
HH32_limb_H3K27me3_r1	HH32_limb_H3K27me3_r2	0.839
HH32_limb_H3K4me1_r1	HH32_limb_H3K4me1_r2	0.323
HH32_whole_H3K27ac_r1	HH32_whole_H3K27ac_r2	0.705
HH32_whole_H3K27me3_r1	HH32_whole_H3K27me3_r2	0.720
HH32_whole_H3K4me1_r1	HH32_whole_H3K4me1_r2	0.621

Supplementary Table 15. The final data set of peaks. The final peaks were chosen from the intersection of the two best ChIP-seq peaks files, and we only kept reproducible peaks that had an average normalized coverage of >=1 in the two replicates.

Sample	Region Number	Total Length(bp)	Average Length	Genome Rate(%)
HH16_whole_H3K27ac	34,189	46,618,268	1,363	4.21
HH16_whole_H3K27me3	10,490	17,957,789	1,711	1.62
HH16_whole_H3K4me1	46,437	48,045,878	1,034	4.33
HH21_limb_H3K27ac	35,770	54,691,279	1,528	4.93
HH21_limb_H3K27me3	13,807	27,144,555	1,965	2.45
HH21_limb_H3K4me1	27,936	23,779,441	851	2.15
HH21_whole_H3K27ac	31,175	40,201,721	1,289	3.63
HH21_whole_H3K27me3	14,191	19,142,269	1,348	1.73
HH21_whole_H3K4me1	59,792	63,819,310	1,067	5.76
HH32_limb_H3K27ac	34,039	44,963,621	1,320	4.06
HH32_limb_H3K27me3	22,507	42,086,868	1,869	3.8
HH32_limb_H3K4me1	28,343	19,724,942	695	1.78
HH32_whole_H3K27ac	27,388	35,175,543	1,284	3.17
HH32_whole_H3K27me3	13,144	25,795,560	1,962	2.33
HH32_whole_H3K4me1	31,924	30,053,746	941	2.71

Supplementary Table 16. Over-representation test for peaks in ASHCEs set using GAT (background: genome).

	Observed length(bp)	Expected length(bp)	Fold	p-value	Percentage in ASHCEs	Percentage in genome
All	2,922,613	1,962,688	1.49	0.00001	26.65	16.99
All H3K27ac	1,853,486	1,118,713	1.66	0.00001	16.90	9.75
All H3K27me3	709,230	560,110	1.27	0.00001	6.47	4.88
All H3K4me1	1,658,184	1,122,162	1.48	0.00001	15.12	9.52

Supplementary Table 17. Over-representation test for peaks (merged peaks of a same histone mark) in different annotation groups in ASHCEs set using GAT (background: genome).

Histone Marks	Genome Annotation	Observed length (bp)	Expected length (bp)	Fold	p-value
H3K27ac	3’ 10kb	147858	94653	**1.56**	0.00001
	5’ 10kb	273040	196878	**1.39**	0.00001
	Exon	2956	1852	**1.60**	0.00109
	Intergenic	826143	443023	**1.86**	0.00001
	Intron	625791	360113	**1.74**	0.00001
H3K27me3	3’ 10kb	65557	53730	**1.22**	0.00002
	5’ 10kb	137851	115188	**1.20**	0.00001
	Exon	1341	1109	**1.21**	0.18499
	Intergenic	345923	274209	**1.26**	0.00001
	Intron	149876	113148	**1.32**	0.00001
H3K4me1	3’ 10kb	140005	103803	**1.35**	0.00001
	5’ 10kb	282635	226987	**1.25**	0.00001
	Exon	3056	2263	**1.35**	0.01500
	Intergenic	690823	419310	**1.65**	0.00001
	Intron	528838	324421	**1.63**	0.00001

Supplementary Table 18. Statistics of chromatin state maps predicted by chromHMM.

		length (bp) in genome	# in genome	length (bp) in ASHCEs
HH16_whole	E1/strong enhancer	62,914,600	56,540	251,452
	E2/weak enhancer	186,004,000	88,683	7,118,635
	E3/low signal	828,370,400	46,076	2,616,047
	E4/poised enhancer	31,983,400	21,173	981,152
HH21_limb	E1/strong enhancer	59,462,000	49,550	364,453
	E2/weak enhancer	103,725,400	66,251	8,229,243
	E3/low signal	910,153,800	43,064	1,427,899
	E4/poised enhancer	36,021,400	19,660	946,083
HH21_whole	E1/strong enhancer	51,714,200	50,679	278,239
	E2/weak enhancer	197,313,400	83,260	7,078,371
	E3/low signal	830,151,000	47,337	2,758,815
	E4/poised enhancer	30,056,200	26,011	851,896
HH32_limb	E1/strong enhancer	55,940,000	57,220	593,232
	E2/weak enhancer	201,542,200	94,837	6,620,541
	E3/low signal	806,212,200	53,857	2,745,530
	E4/poised enhancer	45,631,600	30,086	1,007,608
HH32_whole	E1/strong enhancer	56,620,600	56,521	376,892
	E2/weak enhancer	159,762,600	84,365	7,613,446
	E3/low signal	858,243,200	42,826	2,225,087
	E4/poised enhancer	34,679,400	20,947	751,878

Supplementary Table 19. Over-representation tests for chromHMM chromatin state in ASHCEs using GAT (background: genome).

	Chromatin annotation	Observed length (bp)	Expected length (bp)	Fold	p-value
HH16_whole	E1/strong enhancer	981,152	643,989	1.52	0.001
	E2/weak enhancer	2,614,353	1,904,979	1.37	0.001
	E3/low signal	7,118,635	8,108,414	0.88	0.001
	E4/poised enhancer	251,452	308,811	0.81	0.001
HH21_limb	E1/strong enhancer	946,083	609,204	1.55	0.001
	E2/weak enhancer	1,425,813	1,060,625	1.34	0.001
	E3/low signal	8,229,243	8,938,085	0.92	0.001
	E4/poised enhancer	364,453	358,249	1.02	0.206
HH21_whole	E1/strong enhancer	851,896	525,575	1.62	0.001
	E2/weak enhancer	2,757,086	2,039,067	1.35	0.001
	E3/low signal	7,078,371	8,105,781	0.87	0.001
	E4/poised enhancer	278,239	296,150	0.94	0.002

HH32_limb	E1/strong enhancer	1,007,608	579,431	1.74	0.001
	E2/weak enhancer	2,744,211	2,086,329	1.32	0.001
	E3/low signal	6,620,541	7,829,328	0.85	0.001
	E4/poised enhancer	593,232	470,449	1.26	0.001
HH32_whole	E1/strong enhancer	751,878	574,690	1.31	0.001
	E2/weak enhancer	2,223,376	1,634,235	1.36	0.001
	E3/low signal	7,613,446	8,407,046	0.91	0.001
	E4/poised enhancer	376,892	348,708	1.08	0.001

Supplementary Table 20. Over-representation tests for differential histone modification sites (predicted by diffReps) in ASHCEs set using GAT (background: genome).

	Observed length (bp)	Expected length (bp)	Fold	p-value
H3K4me1_HH21_whole_vs_HH16_whole	260,204	193,138	1.35	0.001
H3K4me1_HH32_limb_vs_HH21_limb	115,386	65,566	1.76	0.001
H3K4me1_HH32_whole_vs_HH16_whole	110,016	70,712	1.56	0.001
H3K4me1_HH32_whole_vs_HH21_whole	206,489	148,797	1.39	0.001
H3K27ac_HH21_whole_vs_HH16_whole	235,317	128,230	1.84	0.001
H3K27ac_HH32_limb_vs_HH21_limb	1,105,783	637,251	1.74	0.001
H3K27ac_HH32_whole_vs_HH16_whole	641,217	394,164	1.63	0.001
H3K27ac_HH32_whole_vs_HH21_whole	542,491	311,389	1.74	0.001
H3K27me3_HH21_whole_vs_HH16_whole	48,928	45,890	1.07	0.108
H3K27me3_HH32_limb_vs_HH21_limb	506,245	384,675	1.32	0.001
H3K27me3_HH32_whole_vs_HH16_whole	148,432	130,923	1.13	0.001
H3K27me3_HH32_whole_vs_HH21_whole	88,128	75,678	1.16	0.001
HH21_H3K4me1_limb_vs_whole	107,299	71,405	1.50	0.001
HH21_H3K27ac_limb_vs_whole	792,640	470,766	1.68	0.001
HH21_H3K27me3_limb_vs_whole	180,352	168,168	1.07	0.004
HH32_H3K4me1_limb_vs_whole	49,078	29,371	1.67	0.001
HH32_H3K27ac_limb_vs_whole	577,339	344,626	1.68	0.001
HH32_H3K27me3_limb_vs_whole	292,522	223,255	1.31	0.001

Supplementary Table 21. Over-representation tests for regions with differential ChromHMM states (eg, E1 to E2/E3/E4) in ASHCEs using GAT (background: genome).

	Observed length (bp) in ASHCEs	Expected length (bp) in ASHCEs	Fold	p-value
HH21 whole vs HH16 whole	2,435,190	1,858,138	1.31	0.001
HH32 whole vs HH16_whole	2,973,456	2,120,811	1.40	0.001
HH32 limb vs HH21 limb	3,459,343	2,493,004	1.39	0.001
HH32 whole vs HH21 whole	2,829,711	2,105,832	1.34	0.001
HH21 limb_vs whole	2,887,412	2,093,827	1.38	0.001
HH32 limb_vs whole	3,226,395	2,393,296	1.35	0.001

Supplementary Table 22. Over-representation test for limb-specific differential sites (up-regulated in limb samples compared to whole embryo samples) in ASHCEs set using *GAT* (background: genome).

Comparison	Stage	Observed length (bp)	Expected length (bp)	Fold	p-value	Percentage of all ASHCEs
H3K27ac_limb_vs_whole	HH21	447,467	286,650	1.56	0.001	4.08
	HH32	413,805	197,254	2.09	0.001	3.77
H3K27me3_limb_vs_whole	HH21	121,738	115,614	1.05	0.080	1.11
	HH32	216,156	130,245	1.66	0.001	1.97
H3K4me1_limb_vs_whole	HH21	34,855	27,756	1.26	0.001	0.32
	HH32	33,125	15,523	2.13	0.001	0.30

Supplementary Table 23. Over-represented TFBSs (including ChIP-seq motifs) of limb-specific differential sites overlapping ASHCEs using *GAT* (background: genome; q-value<0.05).

Motif matrix	TF name	H3K4me1				H3K27ac				H3K27me3			
		Observed Length (bp)	Expected Length (bp)	Fold	q-value	Observed length (bp)	Expected length (bp)	Fold	q-value	Observed length (bp)	Expected length (bp)	Fold	q-value
MA0091.1	TAL1::TCF3	244	51	4.7	0.004	2,908	598	4.9	0.002	478	157	3.0	0.002
MA0119.1	TLX1::NFIC	-	-	-	-	56	12	4.4	0.010	-	-	-	-
De-novo-TAATTAGC	BestGuess: Lhx2(Homeobox)	70	21	3.2	0.004	933	224	4.1	0.002	427	105	4.0	0.002
homer_known_51	Pax7(Paired,Homeobox),longest	-	-	-	-	486	118	4.1	0.002	286	44	6.4	0.002
MA0159.1	RXR::RAR_DR5	-	-	-	-	56	13	4.1	0.007	-	-	-	-
homer_known_44	TCFL2(HMG)	81	18	4.2	0.004	873	220	4.0	0.002	231	75	3.1	0.002
homer_known_22	Mef2d(MADS)	184	49	3.7	0.004	2,366	613	3.9	0.002	1,028	220	4.7	0.002
homer_known_60	NF1:FOXA1(CTF,Forkhead)	80	20	3.8	0.013	897	238	3.8	0.002	367	93	3.9	0.002
MA0052.1	MEF2A	78	17	4.3	0.004	1,000	265	3.8	0.002	465	97	4.8	0.002
MA0009.1	T	-	-	-	-	121	32	3.7	0.002	-	-	-	-
MA0125.1	Nobox	419	128	3.3	0.004	5,446	1,565	3.5	0.002	2,265	600	3.8	0.002
homer_known_58	NFkB-p65-Rel(RHD)	-	-	-	-	383	110	3.4	0.002	142	55	2.5	0.002
homer_known_5	Pax7(Paired,Homeobox),long	-	-	-	-	285	82	3.4	0.002	120	34	3.5	0.002
MA0063.1	Nkx2-5	175	47	3.7	0.004	2,129	621	3.4	0.002	927	218	4.2	0.002
homer_known_27	Pax7(Paired,Homeobox)	81	13	5.8	0.004	825	242	3.4	0.002	300	88	3.4	0.002
homer_known_1	RFX(HTH)	47	14	3.2	0.044	676	202	3.3	0.002	232	83	2.8	0.002
homer_known_19	Tcf3(HMG)	208	61	3.4	0.004	2,208	663	3.3	0.002	697	230	3.0	0.002

MA0046.1	HNF1A	-	-	-	-	166	49	3.3	0.002	60	19	3.1	0.023
homer_known_21	ETS:E-box(ETS,bHLH)	92	25	3.6	0.004	745	225	3.3	0.002	267	100	2.7	0.002
homer_known_3	Hnf1(Homeobox)	170	32	5.1	0.004	1,486	449	3.3	0.002	457	143	3.2	0.002
MA0132.1	Pdx1	402	116	3.4	0.004	4,938	1,498	3.3	0.002	1,904	530	3.6	0.002
homer_known_8	X-box(HTH)	-	-	-	-	992	304	3.3	0.002	346	123	2.8	0.002
MA0137.2	STAT1	56	15	3.6	0.037	499	155	3.2	0.002	150	49	3.0	0.002
homer_known_13	Ets1-distal(ETS)	161	46	3.4	0.004	1,752	547	3.2	0.002	389	167	2.3	0.002
MA0142.1	Pou5f1	-	-	-	-	59	18	3.2	0.024	-	-	-	-
homer_known_20	Rfx2(HTH)	57	16	3.3	0.027	695	223	3.1	0.002	256	87	2.9	0.002
homer_known_45	Hoxb4(Homeobox)	156	50	3.1	0.004	1,693	545	3.1	0.002	719	251	2.9	0.002
homer_known_40	HOXA2(Homeobox)	-	-	-	-	771	249	3.1	0.002	292	137	2.1	0.002
MA0071.1	RORA_1	176	73	2.4	0.004	2,772	903	3.1	0.002	801	345	2.3	0.002
homer_known_17	Nur77(NR)	-	-	-	-	1,396	466	3.0	0.002	513	170	3.0	0.002
homer_known_18	DREF	-	-	-	-	73	24	3.0	0.002	-	-	-	-
MA0151.1	ARID3A	785	225	3.5	0.004	9,753	3,307	2.9	0.002	3,362	1,057	3.2	0.002
MA0099.2	AP1	325	77	4.2	0.004	2,641	899	2.9	0.002	957	343	2.8	0.002
MA0113.1	NR3C1	-	-	-	-	55	18	2.9	0.032	53	8	6.2	0.004
homer_known_29	ISRE(IRF)	51	10	4.8	0.007	382	132	2.9	0.002	-	-	-	-
MA0092.1	Hand1::Tcfe2a	648	209	3.1	0.004	6,586	2,293	2.9	0.002	1,527	821	1.9	0.002
homer_known_39	OCT:OCT(POU,Homeobox)	-	-	-	-	68	23	2.9	0.018	39	9	4.0	0.042
homer_known_28	LXRE(NR),DR4	-	-	-	-	300	106	2.8	0.002	-	-	-	-
homer_known_30	OCT4-SOX2-TCF-NANOG(POU,Homeobox,HMG)	253	54	4.7	0.004	2,131	757	2.8	0.002	681	266	2.6	0.002
MA0047.2	Foxa2	266	54	4.8	0.004	2,110	753	2.8	0.002	765	264	2.9	0.002
homer_known_36	TATA-box	72	17	4.1	0.004	575	205	2.8	0.002	146	59	2.4	0.002
MA0070.1	PBX1	-	-	-	-	305	109	2.8	0.002	-	-	-	-
MA0158.1	HOXA5	304	88	3.4	0.004	3,060	1,100	2.8	0.002	1,121	418	2.7	0.002
homer_known_12	TEAD2(TEA)	349	90	3.9	0.004	3,076	1,114	2.8	0.002	689	389	1.8	0.002
homer_known_42	Nrf2(bZIP)	-	-	-	-	221	80	2.7	0.002	104	36	2.8	0.002
MA0144.1	Stat3	169	46	3.6	0.004	1,287	475	2.7	0.002	426	196	2.2	0.002
MA0090.1	TEAD1	-	-	-	-	956	352	2.7	0.002	248	127	1.9	0.002
homer_known_33	Six1(Homeobox)	120	55	2.2	0.018	1,723	641	2.7	0.002	303	186	1.6	0.008

MA0017.1	NR2F1	-	-	-	-	165	61	2.7	0.002	-	-	-	-
homer_known_25	T1ISRE(IRF)	22	3	6.5	0.032	80	30	2.7	0.007	-	-	-	-
MA0065.2	PPARG::RXRA	-	-	-	-	122	45	2.6	0.009	-	-	-	-
homer_known_23	EFL-1(E2F)	42	5	7.0	0.010	138	52	2.6	0.002	-	-	-	-
MA0030.1	FOXF2	-	-	-	-	467	177	2.6	0.002	185	63	2.9	0.002
homer_known_55	TR4(NR),DR1	-	-	-	-	448	172	2.6	0.002	125	68	1.8	0.044
homer_known_24	NFAT:AP1(RHD,bZIP)	260	71	3.6	0.004	2,248	871	2.6	0.002	703	295	2.4	0.002
MA0136.1	ELF5	557	183	3.0	0.004	6,122	2,379	2.6	0.002	1,895	809	2.3	0.002
homer_known_54	FOXP1(Forkhead)	420	95	4.4	0.004	3,398	1,328	2.6	0.002	1,247	440	2.8	0.002
homer_known_4	IRF2(IRF)	64	19	3.3	0.007	619	242	2.5	0.002	164	96	1.7	0.046
MA0141.1	Esrrb	-	-	-	-	1,085	426	2.5	0.002	446	210	2.1	0.002
MA0114.1	HNF4A	-	-	-	-	777	309	2.5	0.002	-	-	-	-
MA0077.1	SOX9	296	93	3.2	0.004	3,328	1,329	2.5	0.002	1,460	478	3.0	0.002
homer_known_48*	PAX6(Paired,Homeobox)	-	-	-	-	890	356	2.5	0.002	339	165	2.1	0.002
MA0157.1	FOXO3	589	218	2.7	0.004	7,043	2,820	2.5	0.002	2,472	934	2.6	0.002
MA0145.1	Tcfcp2l1	58	17	3.2	0.025	391	156	2.5	0.002	-	-	-	-
MA0108.2	TBP	236	72	3.2	0.004	2,324	939	2.5	0.002	639	320	2.0	0.002
MA0135.1	Lhx3	117	12	9.0	0.004	495	200	2.5	0.002	258	75	3.4	0.002
MA0043.1	HLF	-	-	-	-	928	377	2.5	0.002	233	114	2.0	0.002
MA0102.2	CEBPA	148	85	1.7	0.050	2,599	1,058	2.5	0.002	849	375	2.3	0.002
MA0025.1	NFIL3	-	-	-	-	963	393	2.4	0.002	205	105	1.9	0.002
MA0153.1	HNF1B	36	10	3.5	0.045	317	130	2.4	0.002	212	43	4.8	0.002
MA0087.1	Sox5	459	139	3.3	0.004	4,961	2,048	2.4	0.002	2,027	636	3.2	0.002
MA0150.1	NFE2L2	-	-	-	-	850	366	2.3	0.002	379	174	2.2	0.002
homer_known_35	IRF:BATF(IRF:bZIP)	89	29	3.0	0.015	1,070	461	2.3	0.002	366	135	2.7	0.002
homer_known_46	Rfx5(HTH)	-	-	-	-	1,598	691	2.3	0.002	416	270	1.5	0.006
De-novo-AAATAACAGCGC	BestGuess: Rhox11	127	36	3.5	0.004	771	336	2.3	0.002	340	193	1.8	0.002
homer_known_34	PU.1:IRF8(ETS:IRF)	-	-	-	-	1,141	499	2.3	0.002	307	197	1.6	0.014
homer_known_26	IRF1(IRF)	-	-	-	-	793	350	2.3	0.002	-	-	-	-
MA0160.1	NR4A2	218	106	2.0	0.007	2,496	1,130	2.2	0.002	869	497	1.7	0.002
MA0080.2	SPI1	137	49	2.8	0.004	1,373	627	2.2	0.002	472	207	2.3	0.002
homer_known_57	RAR:RXR(NR),DR5	-	-	-	-	166	76	2.2	0.007	-	-	-	-
MA0164.1	Nr2e3	264	104	2.5	0.004	2,869	1,333	2.2	0.002	1,097	459	2.4	0.002
MA0042.1	FOXI1	447	180	2.5	0.004	5,840	2,717	2.1	0.002	1,953	929	2.1	0.002

MA0152.1	NFATC2	720	280	2.6	0.004	7,485	3,505	2.1	0.002	2,259	1,320	1.7	0.002
MA0038.1	Gfi	777	268	2.9	0.004	6,517	3,058	2.1	0.002	2,766	1,287	2.1	0.002
MA0018.2	CREB1	111	34	3.2	0.004	754	357	2.1	0.002	274	160	1.7	0.004
homer_known_38	OCT:OCT(POU,Homeobox,IR1)	-	-	-	-	138	67	2.0	0.014	90	32	2.7	0.021
homer_known_6	Oct4:Sox17(POU,Homeobox,HMG)	140	42	3.3	0.004	1,142	570	2.0	0.002	400	184	2.2	0.002
MA0101.1	REL	217	125	1.7	0.010	2,227	1,119	2.0	0.002	1,009	661	1.5	0.002
De-novo-AAACAAACGCCG	BestGuess: Sox3	88	36	2.4	0.027	840	426	2.0	0.002	316	216	1.5	0.042
homer_known_56	ZNF317(Zf)	-	-	-	-	501	259	1.9	0.002	193	117	1.6	0.047
MA0002.2	RUNX1	316	195	1.6	0.004	3,828	2,030	1.9	0.002	1,411	915	1.5	0.002
homer_known_59	Dorsal(RHD)	-	-	-	-	905	482	1.9	0.002	514	290	1.8	0.002
MA0019.1	Ddit3::Cebpa	-	-	-	-	1,129	614	1.8	0.002	-	-	-	-
MA0041.1	Foxd3	252	99	2.5	0.004	2,903	1,618	1.8	0.002	1,006	517	1.9	0.002
homer_known_2	GATA:SCL(Zf,bHLH)	-	-	-	-	870	493	1.8	0.002	-	-	-	-
De-novo-TTTAAMCGCG	BestGuess: MBP1::SWI6	83	41	2.0	0.045	681	392	1.7	0.002	456	269	1.7	0.002
homer_known_10	GEI-11(Myb?)	-	-	-	-	161	94	1.7	0.043	-	-	-	-
De-novo-GBTCGTTT	BestGuess: UPC2	-	-	-	-	300	184	1.6	0.002	189	117	1.6	0.018
MA0048.1	NHLH1	-	-	-	-	588	364	1.6	0.002	-	-	-	-
MA0100.1	Myb	118	60	2.0	0.015	961	604	1.6	0.002	-	-	-	-
homer_known_37	FOXA1:AR(Forkhead,NR)	-	-	-	-	561	353	1.6	0.012	-	-	-	-
De-novo-CGGBWWTN	BestGuess: YRR1	-	-	-	-	518	335	1.5	0.002	394	241	1.6	0.002
MA0154.1	EBF1	-	-	-	-	2,321	1,604	1.4	0.002	-	-	-	-
homer_known_47	ETS(ETS)	-	-	-	-	510	378	1.3	0.010	189	93	2.0	0.002
MA0057.1	MZF1_5-13	-	-	-	-	2,433	1,903	1.3	0.002	-	-	-	-
De-novo-GCGGTTTGCTTB	BestGuess: prd	114	46	2.5	0.004	-	-	-	-	-	-	-	-
MA0074.1	RXRA::VDR	15	0	13.9	0.020	-	-	-	-	-	-	-	-

Supplementary Table 24. Enriched GO terms in 'within 10kb' top 500 ASHCEs-associated genes. The GO terms with a FDR adjusted p-value of <0.0001 (chi-squared test) **are shown.**

GO ID	GO term	FDR	#genes

		adjusted p-value	
GO:0009887	organ morphogenesis	1.83E-18	56
GO:0006357	regulation of transcription from RNA polymerase II promoter	1.80E-15	64
GO:0006366	transcription from RNA polymerase II promoter	1.06E-14	65
GO:0045944	positive regulation of transcription from RNA polymerase II promoter	1.41E-14	47
GO:0048513	organ development	3.56E-14	100
GO:0009653	anatomical structure morphogenesis	2.85E-13	88
GO:0045893	positive regulation of transcription, DNA-dependent	3.16E-13	57
GO:0010628	positive regulation of gene expression	4.94E-13	60
GO:0032774	RNA biosynthetic process	4.94E-13	100
GO:0006355	regulation of transcription, DNA-dependent	4.94E-13	97
GO:0048598	embryonic morphogenesis	4.94E-13	39
GO:0009888	tissue development	7.59E-13	63
GO:0006351	transcription, DNA-dependent	7.59E-13	99
GO:0048729	tissue morphogenesis	8.00E-13	37
GO:0060429	epithelium development	1.85E-12	40
GO:2000112	regulation of cellular macromolecule biosynthetic process	2.15E-12	101
GO:0051252	regulation of RNA metabolic process	2.44E-12	98
GO:0009790	embryo development	2.44E-12	55
GO:0010557	positive regulation of macromolecule biosynthetic process	3.18E-12	59
GO:0045935	positive regulation of nucleobase-containing compound metabolic process	3.38E-12	60
GO:0048731	system development	3.63E-12	114
GO:0019219	regulation of nucleobase-containing compound metabolic process	5.60E-12	110
GO:0010468	regulation of gene expression	6.80E-12	107
GO:0007275	multicellular organismal development	9.12E-12	123
GO:0002009	morphogenesis of an epithelium	9.18E-12	31
GO:0035295	tube development	1.65E-11	34
GO:0048856	anatomical structure development	2.23E-11	124
GO:0009889	regulation of biosynthetic process	2.86E-11	104
GO:0031328	positive regulation of cellular biosynthetic process	4.29E-11	60
GO:0031326	regulation of cellular biosynthetic process	4.32E-11	103
GO:0000122	negative regulation of transcription from RNA polymerase II promoter	6.43E-11	33
GO:0007399	nervous system development	1.29E-10	63
GO:0007423	sensory organ development	1.86E-10	33
GO:0032502	developmental process	2.17E-10	132
GO:0022008	neurogenesis	2.28E-10	49
GO:0003700	sequence-specific DNA binding transcription factor	2.35E-10	47

	activity		
GO:0000904	cell morphogenesis involved in differentiation	7.98E-10	32
GO:0048699	generation of neurons	7.98E-10	46
GO:0032501	multicellular organismal process	1.21E-09	143
GO:0045892	negative regulation of transcription, DNA-dependent	1.67E-09	42
GO:0010629	negative regulation of gene expression	1.72E-09	45
GO:0007417	central nervous system development	2.60E-09	36
GO:0048667	cell morphogenesis involved in neuron differentiation	2.65E-09	25
GO:2000113	negative regulation of cellular macromolecule biosynthetic process	2.97E-09	45
GO:0050793	regulation of developmental process	3.27E-09	61
GO:0048522	positive regulation of cellular process	3.48E-09	104
GO:0016070	RNA metabolic process	4.53E-09	105
GO:0035239	tube morphogenesis	4.69E-09	25
GO:0048518	positive regulation of biological process	7.40E-09	111
GO:0043009	chordate embryonic development	7.57E-09	37
GO:0045595	regulation of cell differentiation	9.53E-09	47
GO:0031327	negative regulation of cellular biosynthetic process	1.01E-08	47
GO:0045934	negative regulation of nucleobase-containing compound metabolic process	1.02E-08	45
GO:0051239	regulation of multicellular organismal process	1.20E-08	69
GO:0030182	neuron differentiation	1.21E-08	41
GO:0080090	regulation of primary metabolic process	1.21E-08	122
GO:0010605	negative regulation of macromolecule metabolic process	1.41E-08	53
GO:0031324	negative regulation of cellular metabolic process	1.41E-08	53
GO:0009892	negative regulation of metabolic process	1.44E-08	56
GO:0001501	skeletal system development	1.46E-08	26
GO:0060255	regulation of macromolecule metabolic process	1.52E-08	118
GO:0072358	cardiovascular system development	1.75E-08	41
GO:0048562	embryonic organ morphogenesis	1.84E-08	20
GO:0000902	cell morphogenesis	1.84E-08	41
GO:0044212	transcription regulatory region DNA binding	1.84E-08	24
GO:0031323	regulation of cellular metabolic process	1.98E-08	124
GO:0048812	neuron projection morphogenesis	2.44E-08	24
GO:0010467	gene expression	2.86E-08	116
GO:0010604	positive regulation of macromolecule metabolic process	2.86E-08	65
GO:0048568	embryonic organ development	3.34E-08	26
GO:0060562	epithelial tube morphogenesis	3.60E-08	21
GO:0044249	cellular biosynthetic process	3.71E-08	132
GO:0007420	brain development	4.09E-08	29
GO:0042127	regulation of cell proliferation	4.65E-08	47
GO:0048646	anatomical structure formation involved in morphogenesis	6.37E-08	42
GO:0009058	biosynthetic process	6.68E-08	134

GO:0071363	cellular response to growth factor stimulus	7.24E-08	20
GO:0034645	cellular macromolecule biosynthetic process	7.35E-08	111
GO:0019222	regulation of metabolic process	1.25E-07	132
GO:2000026	regulation of multicellular organismal development	1.54E-07	48
GO:0030154	cell differentiation	1.54E-07	83
GO:0051094	positive regulation of developmental process	1.80E-07	34
GO:0031325	positive regulation of cellular metabolic process	1.91E-07	64
GO:0007409	axonogenesis	1.91E-07	21
GO:0048666	neuron development	1.92E-07	33
GO:0001503	ossification	2.06E-07	20
GO:0008284	positive regulation of cell proliferation	2.62E-07	31
GO:0009893	positive regulation of metabolic process	3.63E-07	67
GO:0021510	spinal cord development	4.57E-07	12
GO:0042221	response to chemical stimulus	5.53E-07	69
GO:0030900	forebrain development	5.87E-07	21
GO:0060021	palate development	6.96E-07	13
GO:0048869	cellular developmental process	8.24E-07	87
GO:0043565	sequence-specific DNA binding	1.47E-06	37
GO:0031175	neuron projection development	1.60E-06	27
GO:0043010	camera-type eye development	1.90E-06	19
GO:0034641	cellular nitrogen compound metabolic process	2.72E-06	141
GO:0090304	nucleic acid metabolic process	2.88E-06	110
GO:0003677	DNA binding	3.53E-06	64
GO:0050794	regulation of cellular process	3.55E-06	196
GO:0048519	negative regulation of biological process	3.84E-06	95
GO:0006935	chemotaxis	4.50E-06	20
GO:0045597	positive regulation of cell differentiation	4.62E-06	26
GO:0060284	regulation of cell development	4.62E-06	26
GO:0007389	pattern specification process	4.82E-06	25
GO:0006139	nucleobase-containing compound metabolic process	4.85E-06	132
GO:0006807	nitrogen compound metabolic process	6.33E-06	142
GO:0048858	cell projection morphogenesis	7.22E-06	26
GO:0005515	protein binding	7.35E-06	274
GO:0003002	regionalization	7.76E-06	21
GO:0048468	cell development	8.05E-06	54
GO:0001655	urogenital system development	8.50E-06	18
GO:0008283	cell proliferation	9.68E-06	51
GO:0001654	eye development	9.98E-06	20
GO:0007411	axon guidance	1.21E-05	15
GO:0050789	regulation of biological process	1.25E-05	202
GO:0060322	head development	1.29E-05	10
GO:0007167	enzyme linked receptor protein signaling pathway	1.33E-05	30
GO:0071495	cellular response to endogenous stimulus	1.58E-05	23

GO:0021915	neural tube development	1.83E-05	16
GO:0044260	cellular macromolecule metabolic process	2.08E-05	167
GO:0050767	regulation of neurogenesis	2.19E-05	21
GO:0007507	heart development	2.55E-05	24
GO:0051960	regulation of nervous system development	2.74E-05	23
GO:0001649	osteoblast differentiation	3.01E-05	15
GO:0016331	morphogenesis of embryonic epithelium	3.05E-05	16
GO:0045664	regulation of neuron differentiation	3.26E-05	18
GO:0005488	binding	3.60E-05	333
GO:0010033	response to organic substance	4.19E-05	45
GO:0070887	cellular response to chemical stimulus	4.19E-05	43
GO:0071310	cellular response to organic substance	4.79E-05	35
GO:0065007	biological regulation	4.91E-05	207
GO:0007610	behavior	5.13E-05	25
GO:0044237	cellular metabolic process	5.53E-05	209
GO:0048523	negative regulation of cellular process	5.60E-05	85
GO:0009719	response to endogenous stimulus	6.02E-05	26
GO:0040011	locomotion	8.29E-05	40
GO:0060173	limb development	8.63E-05	16
GO:0042981	regulation of apoptotic process	9.03E-05	41
GO:0007267	cell-cell signaling	9.38E-05	28

Supplementary Table 25. 16 genes which are associated with the enriched GO "limb development" (GO:0060173).

Gene ID	Transcript ID	Gene Name	Chr	Start	End	Strand
ENSGALG00000001229	ENSGALT00000001864	*SKI*	21	1657223	1747412	-
ENSGALG00000003114	ENSGALT00000004916	*NOG*	18	6194497	6195168	+
ENSGALG00000003324	ENSGALT00000005264	*PRRX1*	8	4978383	5010751	-
ENSGALG00000004368	ENSGALT00000006969	*GNA12*	14	3390192	3424512	-
ENSGALG00000005285	ENSGALT00000008475	*TBX4*	19	7545815	7565605	-
ENSGALG00000005410	ENSGALT00000008687	*WNT5A*	12	8133645	8142169	-
ENSGALG00000007509	ENSGALT00000012140	*GNAS*	20	10870591	10950630	-
ENSGALG00000010529	ENSGALT00000017142	*LEF1*	4	39199281	39268115	+
ENSGALG00000010794	ENSGALT00000017555	*MEOX2*	2	28045101	28098945	-
ENSGALG00000012329	ENSGALT00000020148	*GLI3*	2	50832140	51026884	-
ENSGALG00000014991	ENSGALT00000024179	*HDAC2*	3	67445972	67472193	+
ENSGALG00000016767	ENSGALT00000027071	*AFF3*	1	136801536	136834548	-
ENSGALG00000005396	ENSGALT00000031303	*WNT3A*	2	2364228	2451002	-
ENSGALG00000011630	ENSGALT00000032998	*GLI2*	7	27081494	27526652	+
ENSGALG00000016680	ENSGALT00000036705	*TFAP2B*	3	111026387	111052686	-

ENSGALG00000009723	ENSGALT00000037920	*FMN1*	5	32737307	32859037	+

Supplementary Table 26. Enriched GO terms in 'within 10kb' top 200 ASHCEs-associated genes. The GO terms with a FDR adjusted p-value of <0.05 (chi-squared test) **are shown.**

**GO_ID**	**GO_Term**	**FDR adjusted p-value**	**#genes**
GO:0032774	RNA biosynthetic process	1.34E-05	42
GO:2000112	regulation of cellular macromolecule biosynthetic process	1.34E-05	43
GO:0019219	regulation of nucleobase-containing compound metabolic process	1.34E-05	47
GO:0006355	regulation of transcription, DNA-dependent	1.34E-05	40
GO:0006351	transcription, DNA-dependent	1.34E-05	41
GO:0051252	regulation of RNA metabolic process	1.34E-05	41
GO:0010468	regulation of gene expression	4.30E-05	44
GO:0019222	regulation of metabolic process	4.30E-05	59
GO:0060255	regulation of macromolecule metabolic process	4.30E-05	52
GO:0009887	organ morphogenesis	4.32E-05	20
GO:0006357	regulation of transcription from RNA polymerase II promoter	5.64E-05	24
GO:0031323	regulation of cellular metabolic process	6.24E-05	54
GO:0009888	tissue development	8.56E-05	25
GO:0016070	RNA metabolic process	8.56E-05	45
GO:0080090	regulation of primary metabolic process	0.000133	52
GO:0010628	positive regulation of gene expression	0.000212	23
GO:0010557	positive regulation of macromolecule biosynthetic process	0.000294	23
GO:0044260	cellular macromolecule metabolic process	0.0003	74
GO:0045944	positive regulation of transcription from RNA polymerase II promoter	0.000371	17
GO:0045935	positive regulation of nucleobase-containing compound metabolic process	0.000513	23
GO:0003700	sequence-specific DNA binding transcription factor activity	0.000553	19
GO:0045893	positive regulation of transcription, DNA-dependent	0.000553	21
GO:0048513	organ development	0.000768	36
GO:0090304	nucleic acid metabolic process	0.000778	48
GO:0048522	positive regulation of cellular process	0.000803	42
GO:0035295	tube development	0.000807	15
GO:0048518	positive regulation of biological process	0.000825	45
GO:0034645	cellular macromolecule biosynthetic process	0.001032	46
GO:0031328	positive regulation of cellular biosynthetic process	0.001056	23

GO:0010604	positive regulation of macromolecule metabolic process	0.001131	27
GO:0071363	cellular response to growth factor stimulus	0.001178	9
GO:0051239	regulation of multicellular organismal process	0.001405	28
GO:0010467	gene expression	0.001425	47
GO:0009790	embryo development	0.001628	20
GO:0045595	regulation of cell differentiation	0.001725	19
GO:0005516	calmodulin binding	0.001833	5
GO:0060541	respiratory system development	0.001834	9
GO:0044249	cellular biosynthetic process	0.001834	53
GO:0050794	regulation of cellular process	0.001855	81
GO:0009058	biosynthetic process	0.001948	54
GO:0032502	developmental process	0.002358	50
GO:0007275	multicellular organismal development	0.002892	45
GO:0006139	nucleobase-containing compound metabolic process	0.003403	55
GO:0007167	enzyme linked receptor protein signaling pathway	0.003754	15
GO:0044237	cellular metabolic process	0.004012	87
GO:0050789	regulation of biological process	0.004162	83
GO:0065007	biological regulation	0.004207	86
GO:0043170	macromolecule metabolic process	0.004623	75
GO:0009653	anatomical structure morphogenesis	0.005096	30
GO:0060322	head development	0.005249	5
GO:0050793	regulation of developmental process	0.006144	23
GO:0048598	embryonic morphogenesis	0.007864	14
GO:0042472	inner ear morphogenesis	0.007864	6
GO:0021510	spinal cord development	0.008252	5
GO:0006796	phosphate-containing compound metabolic process	0.009947	28
GO:0031324	negative regulation of cellular metabolic process	0.010089	20
GO:0048856	anatomical structure development	0.010104	44
GO:0008152	metabolic process	0.010854	96
GO:0031325	positive regulation of cellular metabolic process	0.010878	25
GO:0048729	tissue morphogenesis	0.012226	13
GO:0034237	protein kinase A regulatory subunit binding	0.013428	2
GO:0060429	epithelium development	0.013428	14
GO:0060324	face development	0.013829	4
GO:0048646	anatomical structure formation involved in morphogenesis	0.014001	16
GO:0048731	system development	0.01533	39
GO:0005515	protein binding	0.01533	108
GO:0032925	regulation of activin receptor signaling pathway	0.015708	3
GO:0004721	phosphoprotein phosphatase activity	0.01587	7
GO:0032501	multicellular organismal process	0.01587	52
GO:0035239	tube morphogenesis	0.01587	10
GO:0030324	lung development	0.015961	7

GO:0044238	primary metabolic process	0.017036	85
GO:0010033	response to organic substance	0.017402	19
GO:0060021	palate development	0.017551	5
GO:0002009	morphogenesis of an epithelium	0.0177	11
GO:2000113	negative regulation of cellular macromolecule biosynthetic process	0.017902	16
GO:0007423	sensory organ development	0.019496	12
GO:0006470	protein dephosphorylation	0.022013	7
GO:2000026	regulation of multicellular organismal development	0.022741	18
GO:0045934	negative regulation of nucleobase-containing compound metabolic process	0.026132	16
GO:0010605	negative regulation of macromolecule metabolic process	0.026132	19
GO:0008589	regulation of smoothened signaling pathway	0.026167	4
GO:0003677	DNA binding	0.026574	25
GO:0007417	central nervous system development	0.030046	13
GO:0048869	cellular developmental process	0.030708	33
GO:0034261	negative regulation of Ras GTPase activity	0.030999	2
GO:0033504	floor plate development	0.030999	2
GO:0016791	phosphatase activity	0.034135	8
GO:0016311	dephosphorylation	0.034135	9
GO:0021696	cerebellar cortex morphogenesis	0.034135	3
GO:0005488	binding	0.034204	130
GO:0007224	smoothened signaling pathway	0.036275	5
GO:0006464	protein modification process	0.039419	34
GO:0044212	transcription regulatory region DNA binding	0.03985	9
GO:0005634	nucleus	0.041129	54
GO:0060836	lymphatic endothelial cell differentiation	0.041495	2
GO:0010882	regulation of cardiac muscle contraction by calcium ion signaling	0.041495	2
GO:0045892	negative regulation of transcription, DNA-dependent	0.043677	14
GO:0023061	signal release	0.049419	7
GO:0030154	cell differentiation	0.049518	30

Supplementary Table 27. Enriched GO terms in ‘within 10kb’ top 100 ASHCEs-associated genes. The GO terms with a FDR adjusted p-value of <0.05 (chi-squared test) **are shown.**

**GO_ID**	**GO_Term**	**FDR adjusted p-value**	**#genes**
GO:0006355	regulation of transcription, DNA-dependent	5.42E-09	29
GO:0019219	regulation of nucleobase-containing compound metabolic process	5.42E-09	33
GO:2000112	regulation of cellular macromolecule biosynthetic	8.52E-09	30

	process		
GO:0010468	regulation of gene expression	2.80E-08	31
GO:0080090	regulation of primary metabolic process	3.68E-06	34
GO:0060255	regulation of macromolecule metabolic process	4.31E-06	33
GO:0031323	regulation of cellular metabolic process	8.90E-06	34
GO:0034645	cellular macromolecule biosynthetic process	1.44E-05	31
GO:0019222	regulation of metabolic process	1.60E-05	36
GO:0048522	positive regulation of cellular process	2.37E-05	28
GO:0003700	sequence-specific DNA binding transcription factor activity	2.83E-05	16
GO:0048518	positive regulation of biological process	7.74E-05	29
GO:0044249	cellular biosynthetic process	0.000119	34
GO:0006357	regulation of transcription from RNA polymerase II promoter	0.000127	17
GO:0010604	positive regulation of macromolecule metabolic process	0.000165	18
GO:0090304	nucleic acid metabolic process	0.000171	30
GO:0010628	positive regulation of gene expression	0.000458	16
GO:0048583	regulation of response to stimulus	0.000591	21
GO:0006139	nucleobase-containing compound metabolic process	0.00075	34
GO:0048513	organ development	0.000788	22
GO:0044260	cellular macromolecule metabolic process	0.00094	42
GO:0031325	positive regulation of cellular metabolic process	0.00121	17
GO:0050794	regulation of cellular process	0.001379	47
GO:0045944	positive regulation of transcription from RNA polymerase II promoter	0.001385	12
GO:0010557	positive regulation of macromolecule biosynthetic process	0.001792	15
GO:0009790	embryo development	0.002119	14
GO:0045893	positive regulation of transcription, DNA-dependent	0.002259	14
GO:0045935	positive regulation of nucleobase-containing compound metabolic process	0.002259	15
GO:0050789	regulation of biological process	0.002486	48
GO:0008589	regulation of smoothened signaling pathway	0.002883	4
GO:0003677	DNA binding	0.003103	17
GO:0031328	positive regulation of cellular biosynthetic process	0.003305	15
GO:0034237	protein kinase A regulatory subunit binding	0.003556	2
GO:0071363	cellular response to growth factor stimulus	0.003695	6
GO:0065007	biological regulation	0.003695	49
GO:0009887	organ morphogenesis	0.003912	12
GO:0043170	macromolecule metabolic process	0.004675	43
GO:0021510	spinal cord development	0.005238	4
GO:0060541	respiratory system development	0.005238	6
GO:0023051	regulation of signaling	0.005368	18
GO:0035295	tube development	0.005504	9

GO:0007423	sensory organ development	0.005653	9
GO:0043565	sequence-specific DNA binding	0.006295	11
GO:0007275	multicellular organismal development	0.006525	26
GO:0009888	tissue development	0.008777	14
GO:0034261	negative regulation of Ras GTPase activity	0.008777	2
GO:0032502	developmental process	0.011777	28
GO:0060836	lymphatic endothelial cell differentiation	0.012736	2
GO:0048598	embryonic morphogenesis	0.013304	9
GO:0046872	metal ion binding	0.015863	24
GO:0009966	regulation of signal transduction	0.015962	16
GO:0046914	transition metal ion binding	0.015962	18
GO:0008152	metabolic process	0.017213	53
GO:0048731	system development	0.017668	23
GO:0030324	lung development	0.017668	5
GO:0032330	regulation of chondrocyte differentiation	0.017668	3
GO:0032501	multicellular organismal process	0.018821	30
GO:0042472	inner ear morphogenesis	0.018821	4
GO:0021514	ventral spinal cord interneuron differentiation	0.018821	2
GO:0048185	activin binding	0.018821	2
GO:0005516	calmodulin binding	0.018821	3
GO:0005634	nucleus	0.0196	32
GO:0048856	anatomical structure development	0.022749	25
GO:0071310	cellular response to organic substance	0.022815	10
GO:0001946	lymphangiogenesis	0.022912	2
GO:0048584	positive regulation of response to stimulus	0.024823	11
GO:0044238	primary metabolic process	0.026932	47
GO:0048729	tissue morphogenesis	0.027496	8
GO:0010033	response to organic substance	0.027757	12
GO:0060840	artery development	0.031299	3
GO:0002009	morphogenesis of an epithelium	0.031299	7
GO:0010460	positive regulation of heart rate	0.031299	2
GO:0042127	regulation of cell proliferation	0.033701	11
GO:0001047	core promoter binding	0.034474	3
GO:0061298	retina vasculature development in camera-type eye	0.036337	2
GO:0044237	cellular metabolic process	0.03723	46
GO:0023061	signal release	0.039361	5
GO:0009055	electron carrier activity	0.039361	4
GO:2000113	negative regulation of cellular macromolecule biosynthetic process	0.039461	10
GO:0007178	transmembrane receptor protein serine/threonine kinase signaling pathway	0.039461	5
GO:0032332	positive regulation of chondrocyte differentiation	0.039725	2
GO:0021516	dorsal spinal cord development	0.039725	2

GO:0006464	protein modification process	0.041008	20
GO:0008270	zinc ion binding	0.042373	15
GO:0008543	fibroblast growth factor receptor signaling pathway	0.042373	3
GO:0044212	transcription regulatory region DNA binding	0.042385	6
GO:0007167	enzyme linked receptor protein signaling pathway	0.042385	8
GO:0070887	cellular response to chemical stimulus	0.042385	11
GO:0090100	positive regulation of transmembrane receptor protein serine/threonine kinase signaling pathway	0.042385	3
GO:0032925	regulation of activin receptor signaling pathway	0.042385	2
GO:0060841	venous blood vessel development	0.042385	2
GO:0045934	negative regulation of nucleobase-containing compound metabolic process	0.043059	10
GO:0035239	tube morphogenesis	0.044051	6
GO:0008283	cell proliferation	0.044429	12
GO:0005024	transforming growth factor beta-activated receptor activity	0.045029	2
GO:0060429	epithelium development	0.046177	8
GO:0045892	negative regulation of transcription, DNA-dependent	0.049998	9

Supplementary Table 28. Comparison of *dN/dS* ratios between top 500 genes and other genes. Note that some genes don't have *dN/dS* values due to lack of orthologues in Avian Phylogenomics Project.

Group	median *dN/dS* (#genes considered in Top 500)	median *dN/dS* (#genes considered in non-Top 500)	Wilcoxon rank sum test p-value
**Within 10kb**	0.09883 (257)	0.12033 (8036)	0.0486
**5' 10kb**	0.094955 (292)	0.12049 (8001)	0.00072
**3' 10kb**	0.08242 (261)	0.120705 (8032)	0.00016
**Intron**	0.09872 (254)	0.12034 (8039)	0.01108

Supplementary Table 29. Differentially expressed protein-coding genes between chicken phylotypic period (HH16) and other embryonic developmental stages. 'Up' means increased expression level relative to HH16. 'Down' means decreased expression level relative to HH16.

**Comparison**	**>2-fold change**			**>5-fold change**		
	**Up**	**Down**	**Total**	**Up**	**Down**	**Total**
GG_P-vs-GG_HH16	1135	1087	2222	311	500	811
GG_HH6-vs-GG_HH16	651	714	1365	230	318	548
GG_HH11-vs-GG_HH16	312	193	505	86	33	119
GG_HH14-vs-GG_HH16	92	20	112	52	8	60
GG_HH19-vs-GG_HH16	0	0	0	0	0	0
GG_HH28-vs-GG_HH16	970	360	1330	274	34	308
GG_HH38-vs-GG_HH16	1529	1083	2612	530	99	629

Supplementary Table 30. Differentially expressed lncRNA genes between chicken phylotypic period (HH16) and other embryonic developmental stages. 'Up' means increased expression level relative to HH16. 'Down' means decreased expression level relative to HH16.

Comparison	>2-fold change			>5-fold change		
	Up	Down	Total	Up	Down	Total
GG_P-vs-GG_HH16	303	411	714	163	149	312
GG_HH6-vs-GG_HH16	164	239	403	70	88	158
GG_HH11-vs-GG_HH16	39	59	98	18	14	32
GG_HH14-vs-GG_HH16	11	7	18	2	1	3
GG_HH19-vs-GG_HH16	0	0	0	0	0	0
GG_HH28-vs-GG_HH16	199	118	317	64	39	103
GG_HH38-vs-GG_HH16	387	203	590	133	49	182

Supplementary Table 31. High stage-specificity genes in each chicken development stage. '*' indicates the over-representation relative to whole genome background. We considered the genes with tau > 0.5 as high-specificity genes, and considered the stage with highest expression level as the predominantly expressed stage. Furthermore we performed Fisher's exact test to investigate if the top 500 ASHCEs-associated genes have enriched high-specificity expressed genes in these stages

	**Development stage**	**P**	**HH6**	**HH 11**	**HH 14**	**HH 16**	**HH 19**	**HH28**	**HH3 8**
Whole genome (Backgrou nd)	#genes with high specificity (tau > 0.5)	1991	600	457	164	141	189	1014	1661
Top500 gene	#genes with high specificity (tau > 0.5)	50	10	14	7	3	2	51	67
	P-value (Fisher's exact test)	0.95	0.99	0.54	0.25	0.81	0.98	0.00054 1*	0.01 5*

Supplementary Table 32. Differentially expressed protein-coding genes between turtle phylotypic period (TK11) and two late developmental stages (TK15 and TK23, corresponding to HH28 and HH38 in chicken). 'Up' means increased expression level relative to TK11. 'Down' **means decreased expression level relative to TK11.**

Comparison	>2-fold change			>5-fold change		
	Up	Down	Total	Up	Down	Total
PS_TK15-vs-PS_TK11	725	119	844	201	12	213
PS_TK23-vs-PS_TK11	1737	800	2537	786	58	844

Supplementary Table 33. DEGs in the top 500 gene lists (including "within 10kb", "intron", "5' 10kb" and "3' 10kb") showing >5 fold expression level change between phylotypic period (HH16) and later periods (HH28 and HH38). '*' indicates the significant over-representation ($p < 0.1$, Fisher'**s exact test). In total, 90 DEGs (non-redundant) for comparisons of HH28/HH38 vs HH16 are in top 500 gene lists.**

	**#DEGs in given group**	**#expressed in top 500 genes**	**#all DEGs in chicken**	**# all expressed in chicken**	**p-value (Fisher's exact test)**
**GG_HH16-vs-GG_HH28**					
5prime	8	443	308	14902	0.865
3prime	12	441	308	14902	0.3091
intron	21	488	308	14902	0.001963*
within10kb	15	466	308	14902	0.09678*
**GG_HH16-vs-GG_HH38**					
5prime	32	442	629	15122	0.003896*
3prime	28	439	629	15122	0.03033*
intron	30	488	629	15122	0.03943*
within10kb	26	465	629	15122	0.158

Supplementary Table 34. Genes differentially expressed in chicken late stages (HH28 and HH38) relative to the phylotypic period, but not differentially expressed in corresponding turtle stages (TK15 and TK23). The *dN/dS* ratios were calculated based on chicken-turtle orthologs from Ensembl. 'up', up-regulated; 'non', non-differentially expressed. The *dN/dS* ratios were **calculated based on chicken-turtle orthologs from Ensembl.**

**HH28 vs HH16 and TK15 vs TK11**				
**Chicken Gene ID**	**Gene name**	**HH28 vs HH16**	**TK15 vs TK11**	***dN/dS***
ENSGALG00000006627	*UPB1*	up	non	0.08272
ENSGALG00000011099	*FAP*	up	non	0.13988
ENSGALG00000012834	*AKR1D1*	up	non	0.05925
ENSGALG00000019280	*LMO3*	up	non	0.01548
ENSGALG00000023793	*Unknown*	up	non	0.31510
**HH38 vs HH16 and TK23 vs TK11**				
**Chicken Gene ID**	**Gene name**	**HH38 vs HH16**	**TK23 vs TK11**	***dN/dS***
ENSGALG00000001869	*LINGO2*	up	non	0.07168
ENSGALG00000003903	*Unknown*	up	non	0.03954
ENSGALG00000004790	*WNT4*	down	non	0.01865
ENSGALG00000006665	*GALNT6*	down	non	0.03029

ENSGALG00000006726	*GATA3*	up	non	0.02629
ENSGALG00000007636	*PCK1*	up	non	0.08164
ENSGALG00000011809	*GRIN2B*	up	non	0.02025
ENSGALG00000015598	*RASGRF2*	up	non	0.03718
ENSGALG00000023793	*Unknown*	up	non	0.31510

Supplementary Table 35. The primers for cloning. Genes examined in chicken and mouse are shown in red, and those examined in chicken, mouse and gecko are highlighted in blue.

Gene name (NCBI official symbol)	Chicken forward primer	Chicken reverse primer	Mouse forward primer	Mouse reverse primer	Gecko forward primet	Gecko reverse primer
**Rps6kc1**	GCTGTTGGCCTTTGCCTTAC	AAAGCTCTGTTCGGGACTCG				
**Kiaa1328**	ATGACTCGTGACTGTGCCTG	ATGCTGGACTGGTGATGTGG				
**Inadl**	TGTCTTACCACCCCCATTGC	ACCGCTGGCCTGAATCATAG	AGCTCTCACCTACTGGCAGA	GACGATGGGCTATCTTGCGA	CAYGARGTNTAYGARGARGG	GCYTGDATCATNGCDATRAA
**Dach2**	TGAAGGTGGCCTCGTTTCTC	CTGCCCTGGAAAGAGGACTG	CTTGCTGCTTCTGGACCTCA	CAACTGCAAGTTGTCGCTCC	ATGAARYTNATGGCNATGAA	TGYTGDATYTGYTTYTCYTG
**Tbl1x**	GAACGGAGAAGCCACTGTGA	TTGGAGTTTGGGTTGCTGGT	TCCACCGAGTAAAGCCACAG	CCCACAGTCGAACCGTAGAG		
**Lsamp**	GTCCGCAGCGTGGATTTTAC	CTCCAGACCATTGGCACTGT	CACCATGGTCGGGAGAGTTC	TTGAGGGAAGCTTGTCGTCC		
**ENSGALP00000023066**	not cloned					
**Rnf220**	GCAAAGAGAGAAGGCTCATGC	CTGGGGACAGAGCTTCTTGG	TTCCCTTCACCAATGGCTCC	GAGCATTCAGTCGGGTTTGC	TTYGARGARTAYGARTGGTG	ARCCARCAYTCYTCRCARTG
**Tbc1d5**	CTTGTTGTCTGGGACGCTCT	TCAGCTTCCAGTTGGCTCTG				
**Btbd7**	AGCAAGGACCACCTGCTTAC	AGTTGCTGGTGGGTGGTATG				
**Camk2a**	TGAAGCCTGAGAACCTGCTG	GGGGTCACACATCTTCGTGT				

**Mllt10**	GAGAACCCGCTCGTCTACTG	CCAGGCTTTCTTCCCCTCTG				
**Ppp3cb**	TTTGATTGCCTCCCTCTTGCT	AACCCTTGAGTGTCAGCACA				
**Inhba**	GTTCAGTCGCTGACTGTCCA	AGGAAGGGCCTATGCGATTG	GACTTTTGCTGCCAGGATGC	GATGAGGGTGGTCTTCGGAC		
**Fgf18**	GAGAACCAAACGCGAGCAAG	TTAACTGGGGTTGGTGGGTC	CCTGCACTTGCCTGTGTTTAC	TTCTCGCAGTTTCCTCGTTCA		
**ENSGALP00000037769**	not cloned					
**Etfa**	TCCTGTTTGGTTGCTGGGAC	GCCAAGTGTTGGATTGCTCC				
**Gtdc1**	TTTCATGCAAACGGTGCCAG	CCTGTGAGGCCAGACAACAT				
**Psmb1**	TGGCTCTATTCGCACCTTGT	CCTTCTCCAGGGTCAGAGGT	TAGGCGCGATGCTTTCCAC	ACCCGCATGTCAGTCTTTCC		
**Ikzf2**	GCTGAGGCACATCAAGTTGC	AAGCCTTGGCATCCTCCTTC	GCCGTGAGGATGAGATCAGG	GGGGTCGACTCTTTGGTCTG		
**Zmym2**	CACACAAACCCGCTCCAAAG	TCCATCAGGCATTGTGGCTT				
**ENSGALP00000037219**	not cloned					
**Zbtb49**	ACCATGGGTGTGACGAATGT	CTTGTGCCGCCGTAAAACAG				
**Tbr1**	ATGCAGCTGGAGCATTGTCT	ATGCTGGGGTGTCGTAGTTG				
**Smyd2**	TGGCCAATGCATAAGCTGGA	TTTTTGCCCGTAACGCAGTG				
**Spry2**	CAGAGCTATCCGCAACACGA	CGATTGACCCGGTCGTAACA	GTTCCAACGGTGGAGGACTG	AGGCATGCAGACCCAAATCA		
**Ptprk**	ACTTGCAAAGAAGCGCAAGG	AAAGCAGGCCTGTTGCATTG				

**ENSGALP 00000033 157**	not cloned					
**Ahsg**	ACATGCAGTT GAGGGTGACT	ATCTTACCAG GGCACAGTG G				
**Edn2**	GTGTCCTCCT CGAAGATGGT ATG	CTTCTTCCAA GGCAAAACTG CT				
**ENSGALP 00000038 023**	not cloned					
**Fam172a**	ACGGTAATGG CGTTGTCAGA	ACCGAGCTGT TCTTTGCCTC				
**Fam53a**	CTGCCTACCC TTTCCCAGTG	GACTGGAACC AGGGGTGAT G	AGCCACTTT CCTGCGTAG AC	CCTGAGCTT GTACTGCCC TC		
**Bmper**	GTGCTTGCTT CCTGGAGAGT	TCGCTTGTGC CCATTGTAGT	CCCACCCTG GTGTCTGTA AC	TGTAGAGTG GCAGGTCTG GA		
**ENSGALP 00000038 857**	not cloned					
**Itpr1**	CTTCACAGAC CCGAACTGCT	AAGAAGCGG AGGATGGGTT G				
**Cyp3a7**	TGGAAGCTAC AGCATGGACG	ACCTGGAAG GTGAAGTGCT G				
**Pbx3**	TGACCATCAC CGACCAAAGC	ACTTGGGACC CCTGGTAAGA	CGTCCCATT TCCCCGAAA GA	AAGCGCCG GGGATTAGT TAG	TGYGARATH AARGARAAR AC	TTNGCYTCY TCYTGRAAY TT
**Sim1**	CAGCGCAGTG ACAGAGATCA	TGTCCTCTCC CGTGAATCCT	GAAGAAAGG GAGCCCGAG AC	ACGATGGTG GCGTAACTC TG	AAYATGTTYA TGTTYMGNG C	TCYTCYTTD ATCATYTGY TG
**Elp3**	TCGGTACGAC CCCTATCTCC	CGGGTCTTCG TAGGAGAGGA				
**Arhgap15**	CAACTTCTGA CACGGCCTCT	CACAAACTGG GGGACGGTA G				
**Cadps**	AGATTGTGGC AGACGAAGCA	GACTTCAGAC CCTGCACCTC				
**Arfgef1**	AAGCAAAGTC CCCCTCATGG	GGCTTGGTCA GCTTCTGTCT				

**Prkar1b**	AACCAGACCGACCCATGAAG	AACCTCGACGTACTCCTCGT				
**Mprip**	CGTGTCTGCATCTCCACTGT	CCACCGTGTCAACCATCTCA				
**Aldh1a3**	CAGCAAAGGCAGCATTCCAG	ATGGCAAGACCACCACACTC	TTATGGCTACCACCAACGGG	TCAGCATCTGCACACACGAT		
**Lpcat2**	CGTTCCCAATCCCTTCGTCT	TTGCCATGGTTTGTCGGACT				
**Prrx1**	TTTTCCGTGAGTCACCTGCT	CTGTGGGCACTTGATTCCTCT	ACAAAGCCTCCTCTCCATGC	TCTAGCAGGTGACTGACGGA		
**Mbip**	CACATCAGCACTTCAGCCAAC	CCCTGGACTTGCTTTCCTTTG				
**Nt5c2**	GAGCCGCATGAACGAAGTTG	CGGCTCTGAAGAGGTAGCTG				
**Ebf1**	TTTCCACTTCGTCCTGGCTC	ATCTTCCAGGCGTTCCCTTG	TATGTGCGCCTCATCGACTC	TTCACACCCATCATCCCTGC		
**Wdr33**	AGACCACGGGGGATATGTGA	TCCCAGGCAGCAGATTCAAG				
**Agpat3**	GAGTGCACCTTGTTCTCCGA	GCTCCGACAAATCCCAGGAA				
**Bcl7a**	AAAGTGCGCAAATGGGAGAAG	TGTTTTGTTGGGAAGCCTCTAC				
**Mpped1**	GCAGCTAGAAGGCATCAGCA	AGCCTTGGCTGTATTCGTCT	ACCCAGGCCTTCACCTTCTA	TTCTACTCGTTGCTGGCTGG	not cloned	
**ENSGALP00000034681**	not cloned					
**ENSGALP00000034090**	not cloned					
**Pck1**	GCTCTTCGAATTGCCAGCAG	GCACACGGGAATTCTCTCCA				
**Mesdc1**	TTGTCTGTCTTCGGCGAGTC	TTGGGTCAGGAGCACACAAG	TAAGGGGCTGTCCATCCTCA	GCGGTAAAGTCCTGGGTGAA		

**C8h1orf168**	CCAAAGCAGCCAGTGTCCTA	CTGCCTCATATGCGACACCT				
**Arhgap8**	CCCCTCCTACCAGCTCAATC	AGCACTCAGAGAGGCTGTTC				
**ENSGALP00000029750**	CTGTCTGGTGTGATGCTCTGT	TGATCAGTTACAATCTCAGCCCT				
**Cdc42bpa**	CCTGAGTGTGACTGGTGGTC	TGGAAGCATCGTCCAACTCC				
**Dpyd**	CTGGGTTTTTGCAGGTGGTG	GCAGAGTCAATGCCTCCAGT	TCGAGCTGTTCCAGAGGAGA	AGCTTTGTCCAGGGCCATAC	AAYTTKGAYGAYATHAARCA	TCCATYTCYTCNGGNACNGC
**Stx17**	AGCTCCGTCGAATTGAACCT	CCCACCACTCCTCCAATGAC				
**Gpr125**	CCGCAATTATGGCAGCAGAC	GGTGCCCATTTGTCCGAAAC				
**Zak**	AAGGCGATGCTTCTGGTGAA	CTGTCCCTTTCTGAAGCGGT				
**Boc**	GGGCACAACAACCGAGTCTA	AGCAAGGCATTGGTCTCCTC	GACGGCGGTATCCCTACTTG	AATGCTGGACCCATCCTTGG	TAYCARTGYATGGCNGARAA	AYRTACATCCAYTTNARCAT
**Kpna1**	CCTGCTGGGCTTTGTCCTAT	CCTGTGGGGCAATGCTACTA				
**Map3k4**	ATTGGCCAAGTTTGCGACAC	CCCTCCGGACTCACTTTGTC				
**Exoc5**	TGTTCGAGGAGCCCTTTGTG	TGGCTAGCACAGTCTCTGGA				
**Map3k7**	TCCGCATAATGTGGGCAGTT	GTGCCATTTTGCAGTGCTGT	CCCAAGGAGTGGCTTACCTG	CATCCTCTTGCCCTCGGAAG	GARGARATHGAYTAYAARG	CRTTRTGNACNGCCCACAT
**Pde8a**	CGGATAGCACAGGCAATGGA	AGAGATACGTCCAGCCCACT				
**Spag6**	TTGCTGGATGTGGTCCCAAC	GTGAAAGCTCAGGCGAATGC				
**Hgf**	CTGATGGCAAGCTGAGACCA	ATTCCGAACTTGCCTGTGCT				

**Ptprg**	CGCCAGAACGAGAGAACAGT	TCACGACTTGGCCAGTACAC	CTCAGCCTTGACCTTCGTGT	GATCACCGTCCGCTCATTCT		
**Ptpn2**	GCGCGCTATCTGGAAATTCG	GAGACTGCTCACTGCTTGGT				
**Ppp1r3a**	CAGGACCTGGGGCTTACTTG	CGATGCTTCTGGGCACTTTG				
**Pax9**	AGCAAAATCCTGGCTCGCTA	GGAGGGTCACAGCCATTAGG	CGTGTGCGACAAGTACAACG	TGTGACGAGAGGGAGGATGT	ATGGARCCNGCNTTYGGNGA	GGNACRTTRTAYTTRTCRCA
**Fbln1**	GAATTTGCCAGCGTGCATGA	GGTGAGATTCGCTGACAGGT	CCGAGACCAGACCTGTGAAC	TAGAAGCCAGCCTTGCACTC		
**Nsun6**	GGTTTGAATGGCCGAACGAC	TTCCTTGCAAAGTCCCAGCA				
**P2rx1**	CAAGGGCCTGACAATGACCA	TTCGAAGGCTCCTGTTCTGC				
**Errfi1**	TCATTAACCGCAGGCTGTGT	CACCAGACCAGGACTGAACC				
**Tfap2c**	ACGGTCATTAGAAAAGGTCCCA	CTCTGGTCGCCTGTGTTCAT	GTCACCACCGGAATGCCTAA	AGCAGCTAAGCGTGGAGTTT		
**Slc4a10**	ACCAGCCGATACTTCCCAAC	GTCCTTCTAGCGGCAACTGA				
**Ppp6r3**	TGCTCAGGCTACTGACTTGC	ATGTTTCCCACCGCTCTCTG				
**Pemt**	GCCTGGATTACAGCGAGGTG	TGCTCCCTTCTGTTTCTGACG				
**Papola**	TTCCACACGGATGGTCATGG	ATGGTAGGCTTTGGTGGTGG	TAGAACGTTGCTGTGGTAGCG	CATAGCCCAGGAAACACCAC		
**Cul4b**	TGTCCAGTTTCGCCAACAGT	TTGCACTCCACCTCTCACAC				
**Son**	GGCGACCTCGGAATCTGTAG	CCTCTCACTTCTGCAACGGT	TACCGTGGCAGTGTCAGAAC	GCCTGGGCACTCTCTTTGAT		
**Nrcam**	TCCTGTTGGCTTACCACCAC	CAGCACTTGACCGTTCTTGC	GAGGGGAAGGCAGAGACCTA	AAATGGTGTGATGGACGGCT		

**Uchl3**	CCCCGACGTC ACCAATCAG	TAAGCTGCAG ACAGTGCGAT T				
**Hmgcll1**	AAGGAGCAGC TGTGGATCG	AGGATATTGG CTAGCGCCTG				
**C26h6orf1 06**	CTCCTCGGCT TCCAGCTTAG	TCCGAAGGG ATAAGGACCG T				
**Lef1**	TCAAGTCCTC GCTGGTCAAC	GTCATTCTGG GGCCTGTACC	CGTCCTCTC AGGAGCCCT AC	GCCATAATT GTCTCGCGC TG		
**ENSGALP 00000039 242**	CTCCATGGCT GATGGGATGC	GAATCCTCCC TGGCTGAGAC				
**Cyp3a4**	ATGGCCCTAC CAGACCTTCA	TGCACGTCAG GGTGTATAGC				
**Azin1**	TGGGAACCCT TGGGATTGGA	CACAGGATGG ACCCCAAAGG				
**Ebag9**	CCATAACACA GTTTCGGCTC T	TCGTTGTTGT TCTGCTGCTC T				
**Thrap3**	GCTCGTCAGG TTTGAGTCCA	CTCAGAGGC CTTCCTTGGT G				

Supplementary Table 36. The primers for reporter assay. Lower case letters in the sequence column indicate restriction enzyme recognition sequences.

Primer name	Sequence	Target	Remarks
**ASHCE-left-arm-F (EcoRI)**	GGGTTgaattcTGGTT CAGGAACATGATAA G	Sim1 ASHCE 5 kb left arm	for retrieving
**ASHCE-left-arm-R (BamHI)**	GGTggatccTGTAATA TATTATGCTTCTG	Sim1 ASHCE 5 kb left arm	for retrieving
**ASHCE-right-arm-F (BamHI)**	GGTggatccGAAAGC TGGAGAGCTCGTTT	Sim1 ASHCE 5 kb right arm	for retrieving
**ASHCE-right-arm-R (HindIII)**	GGTaagcttGTGCTTT CCAGACAGATGAA	Sim1 ASHCE 5 kb right arm	for retrieving
**5'-Sim1-LA-LacZ**	GATGAAGCTCCGGA GCTTAAACTGTAGG AAGGAAGGGGGAA AAAAACACGATGGC TCGCGATGATCCCG T	LacZ with holology arms for Sim1	for BAC modification
**3'-Sim1-RA-PS**	AGCGGCACCTGGAT GGGAAGGGGAGCG CGGAGGAAAGCCG	LacZ with holology arms for Sim1	for BAC modification

	TCCCACCTACGTGTAGGCTGGAGCTGCTTC		
**Sim1 ASHCE 1 kb-F (SalI)**	gtcgacAGATCCAGCTGGCACACAAG	Sim1 ASHCE 1 kb	for Tg mouse
**Sim1 ASHCE 1 kb-R (HindIII)**	aagcttGGAACCAGAGCTCCTAGTGG	Sim1 ASHCE 1 kb	for Tg mouse
**Sim1 ASHCE 1 kb-F (XhoI)**	TTActcgagAGATCCAGCTGGCACACAAG	Sim1 ASHCE 1 kb	for chicken electroporation
**Sim1 ASHCE 1 kb-R (EcoRV)**	TTgatatcGGAACCAGAGCTCCTAGTGG	Sim1 ASHCE 1 kb	for chicken electroporation
**Sim1 ASHCE 284 b-F (SalI)**	GGTgtcgacCAACTGTGGAAAGAATGGCT	Sim1 ASHCE 284 b	
**Sim1 ASHCE 284 b-R (HindIII)**	GGGaagcttTGTACATCTATCTGTACGAG	Sim1 ASHCE 284 b	
**Sim1 ASHCE upstream 2.5 kb-F (SalI)**	gtcgacTGGTTCAGGAACATGATAAG	Sim1 ASHCE upstream 2.5 kb	
**Sim1 ASHCE upstream 2.5 kb-R (SpeI)**	actagtGATGGAAATTCTAGGTTCTC	Sim1 ASHCE upstream 2.5 kb	
**Sim1 ASHCE downstream 2.5 kb-F (SpeI)**	actagtTTATATGGATCCTGAAGGTG	Sim1 ASHCE downstream 2.5 kb	
**Sim1 ASHCE downstream 2.5 kb-R (HindIII)**	aagcttGTGCTTTCCAGACAGATGAA	Sim1 ASHCE downstream 2.5 kb	
**LacZ 3F**	TCACCCTGCCATAAAGAAACT	LacZ	for genotyping
**LacZ 3R**	CTGTCGTCGTCCCCTCAAACT	LacZ	for genotyping
**BAC_T7F**	GCGCGCCAATAGTCATGC	BAC T7 sequence	for genotyping
**BAC_T7R**	TAGCGGCCGCAAATTTATTA	BAC T7 sequence	for genotyping
**BAC_SP6F**	GCTGCAGATCCCTAAACAGC	BAC SP6 sequence	for genotyping
**BAC_SP6R**	CGGATTTTTCCGTCAGATGT	BAC SP6 sequence	for genotyping

Supplementary References

1 Wang, Z. *et al.* The draft genomes of soft-shell turtle and green sea turtle yield insights into the development and evolution of the turtle-specific body plan. *Nature genetics* **45**, 701-706, doi:10.1038/ng.2615 (2013).

2 Jarvis, E. D. *et al.* Whole-genome analyses resolve early branches in the tree of life of modern birds. *Science* **346**, 1320-1331, doi:10.1126/science.1253451 (2014).

3 Hubisz, M. J., Pollard, K. S. & Siepel, A. PHAST and RPHAST: phylogenetic analysis with space/time models. *Briefings in Bioinformatics* **12**, 41-51 (2011).
